# Role of g5Rp in African swine fever virus replication: disruption of host translation and autophagy

**DOI:** 10.1128/jvi.01252-25

**Published:** 2025-12-15

**Authors:** Chunmei Xu, Ruiying Liang, Yongqiang Zhang, Xinyue Zhang, Xiangyin Zhang, Xinru Luo, Dahu Liu, Shaohua Hou, Jiabo Ding, Xinming Tang, Lin Liang, Lingling Chang, Jinming Li, Changjiang Weng, Zhiliang Wang, Xiaomin Zhao

**Affiliations:** 1 College of Veterinary Medicine, Northwest A&F University 12469, Yangling, Shaanxi, China; 2 Engineering Research Center of Efficient New Vaccines for Animals, Ministry of Education, Yangling, China; 3 Key Laboratory of Animal Biosafety Risk Prevention and Control (North) & Key Laboratory of Veterinary Biological and Chemical Drugs of MARA, Institute of Animal Science, Chinese Academy of Agricultural Sciences 243826, Beijing, China; 4 China Animal Health and Epidemiology Center 499141, Qingdao, Shandong, China; 5 Division of Fundamental Immunology, National African Swine Fever Para-reference Laboratory, State Key Laboratory for Animal Disease Control and Prevention, Harbin Veterinary Research Institute, Chinese Academy of Agricultural Sciences 111613 , Harbin, Heilongjiang, China; College of Agriculture & Life Sciences, University of Arizona, Tucson, Arizona, USA

**Keywords:** ASFV, g5Rp, eIF5A, RPS15, 9″-methyl salvianolate B

## Abstract

African swine fever (ASF), caused by the African swine fever virus (ASFV), is one of the most severe viral diseases affecting swine. ASFV employs sophisticated strategies to subvert host immune responses; however, the function of the viral protein g5Rp in viral pathogenesis remains incompletely defined. In this study, we demonstrate that g5Rp plays a critical role in viral replication by impairing host translation and autophagy. Overexpression of g5Rp enhanced viral replication and increased p30 protein levels, whereas siRNA-mediated knockdown of g5Rp suppressed both, underscoring its essential proviral function. Proteomic profiling of infected porcine macrophages (3D4/21 cells) revealed that g5Rp dysregulated 122 host proteins, predominantly involved in translation, autophagy, and apoptosis pathways. Mechanistically, g5Rp directly interacted with eIF5A and RPS15, disrupting their complex formation and thereby inhibiting translation initiation and autophagic flux. Structural analyses identified key residues (SER¹¹⁸, SER²⁰⁶, and ASN⁶¹) critical for this interference. Mutation of these residues abrogated g5Rp activity. Furthermore, virtual screening identified 9″-methyl salvianolate B as a potent g5Rp inhibitor, which restored eIF5A hypusination, promoted autophagy, and suppressed ASFV replication *in vitro*. Collectively, our findings establish g5Rp as a pivotal regulator of ASFV pathogenesis and a promising target for antiviral drug development.

**IMPORTANCE:**

ASFV has caused significant economic losses to the global pork industry, and no effective treatment or prevention currently exists. In this study, the interaction of g5Rp with the host proteins eIF5A and RPS15 was identified for the first time, and its crucial role in the viral life cycle was clarified. Resolving the crystal structure of g5Rp revealed its binding site to the host protein, which provides a new target for developing antiviral strategies against g5Rp. Additionally, the screened 9″-methyl salvianolate B, a small-molecule inhibitor, has shown the potential to effectively reduce viral replication and restore host protein synthesis. These findings not only deepen our understanding of the mechanism of ASFV infection but also lay the foundation for developing effective anti-ASFV treatment strategies in the future, which has important scientific implications.

## INTRODUCTION

African swine fever virus (ASFV), the causative agent of a lethal hemorrhagic disease with near 100% mortality, poses a severe global threat to the swine industry (1, 2). Despite its identification over a century ago (3), effective vaccines or antivirals remain unavailable, underscoring the imperative to decipher viral strategies for host defense evasions (4, 5).

The g5R protein (g5Rp), ASFV’s sole decapping enzyme, harbors a Nudix motif that hydrolyzes the mRNA 5′ cap structure (releasing m⁷GDP) upon RNA binding (6, 7). Expressed in the endoplasmic reticulum during early infection and accumulating progressively, g5Rp cleaves the 5′ cap moiety to facilitate mRNA destabilization, thereby promoting host cellular shutoff and viral replication (6–9). However, a critical limitation challenges the physiological relevance of this mechanism. Current characterization of g5Rp’s decapping activity relies exclusively on its cleavage of cap 0 (m⁷GpppN), using actin mRNA as the sole substrate (7). This contrasts starkly with the natural context: both mammalian and ASFV mRNAs predominantly utilize cap 1 (m⁷GpppNm) structures, with ASFV transcripts overwhelmingly (92%) bearing cap 1 modifications (10–12). Thus, ASFV likely employs specialized molecular mechanisms, distinct from mRNA decapping, to effectively inhibit host protein synthesis.

Recently, host-pathogen interaction omics studies have revealed how viruses hijack cellular machines through molecular mimicry. Specifically, DNA viruses frequently target ribosomal components and translational initiation factors to preferentially synthesize viral proteins. For example, ASFV pCP312R interacts with the host RPS27A, inhibits host protein translation, and promotes viral replication (13). The Epstein–Barr virus vDUB hindered the Ubiquitin-fold Modifier 1 Modification (UFMylation) of RPL26 and inhibited reticulophagy (14). ASFV UBCv1 binds to eIF4G1 and RPS23 to regulate host translation (15). Recent studies have shown that ASFV pE66L, MGF110-7L, EP152R, and RNA polymerase subunits C315R and H359L induce the closure of host translation via the PKR/eIF2α pathway (16–19).

Autophagy is a conserved “clearance” mechanism in eukaryotic cells that maintains cellular homeostasis and orderly life activities by degrading and recycling intracellular components. Viral infections frequently induce autophagy to degrade viral components and virions. During ASFV infection, many viral proteins interact with the host proteins to regulate autophagy. For example, the ASFV MGF300-4L protein is associated with viral pathogenicity by promoting the autophagic degradation of IKKβ and improving the stability of IκBα (20). ASFV MGF_360-4L inhibits interferon signaling by recruiting the mitochondria-selective autophagy receptor SQSTM1, thereby degrading antagonistic innate immune responses (21). ASFV p17 facilitates mitophagy by promoting the interaction between SQSTM1 and TOMM70, which in turn regulates innate immunity (22). ASFV E199L promotes autophagy by interacting with PYCR2 (23). ASFV K205R induces ER stress, which activates autophagy and the NF-κB signaling pathway (24). However, it remains unclear whether ASFVg5Rp regulates autophagy.

In this study, we explored a novel function of ASFV g5Rp and found that g5Rp interacts with eukaryotic translation initiation factor 5A (eIF5A) and ribosomal protein S15 (RPS15) to reduce host protein synthesis. It also downregulates eIF5A and inhibits TFEB, thus suppressing autophagy. Additionally, a small-molecule inhibitor targeting g5Rp, 9″-methyl salvianolate B, effectively alleviated g5Rp-induced inhibition of protein synthesis and autophagy. Our data elucidate multiple regulatory functions of g5Rp during ASFV replication, showing that g5Rp can evade host immune surveillance and attenuate the host cell response to viral infection by inhibiting autophagy.

## RESULTS

### g5Rp promoted ASFV replication and modulated host pathways

**Fig 1:**
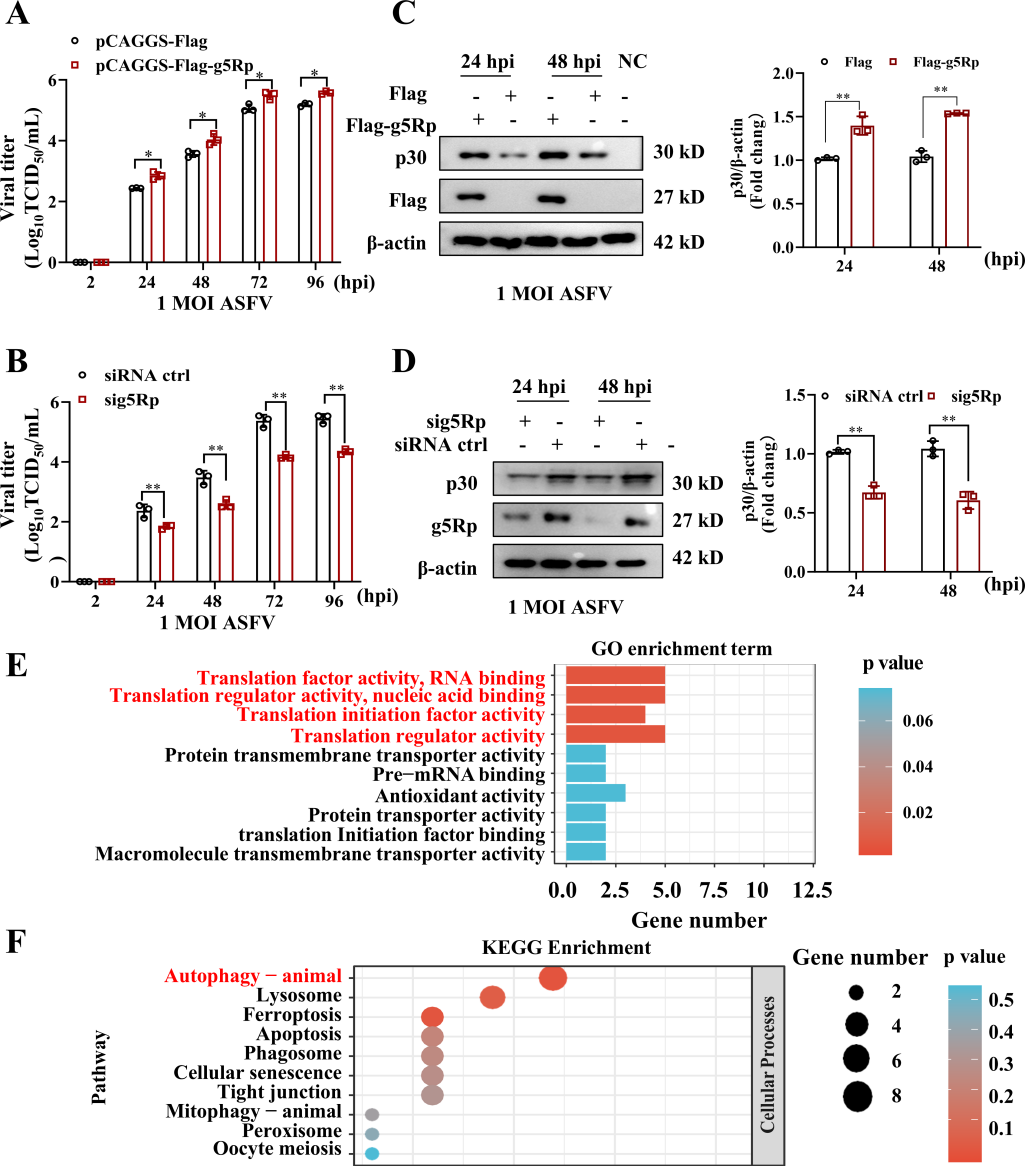
g5Rp promotes ASFV replication and remodels host pathways. (**A**) g5Rp overexpression enhances ASFV replication. Viral titers (TCID₅₀) in cells transfected with either the control plasmid (pCAGGS-Flag) or the g5Rp-expressing plasmid (pCAGGS-Flag-g5Rp) following ASFV infection (MOI = 1). (**B**) g5Rp knockdown inhibits ASFV replication: viral titers (TCID₅₀) in cells transfected with control siRNA or g5Rp-targeting siRNA (sig5Rp) following ASFV infection (MOI = 1). (**C**) g5Rp overexpression increased ASFV p30 protein levels. (**D**) g5Rp knockdown decreased ASFV p30 protein levels. (**E**) and (**F**) GO enrichment analysis and KEGG pathway enrichment. Data are represented as mean ± SD from three independent experiments. Statistical significance was determined using Student’s *t*-test: *, *P* < 0.05; **, *P* < 0.01.

The g5Rp protein facilitates ASFV replication and influences various host cellular pathways. To elucidate the role of g5Rp in ASFV replication, we first examined the dynamics of endogenous g5Rp expression. Low expression levels during the first 0–4 h post-infection (hpi) (Fig. S1A) suggested minimal involvement in the early stages of infection. Based on this observation, we constructed a pCAGGS-Flag-g5Rp overexpression plasmid. Overexpression of g5Rp enhanced viral replication and increased p30 expression (Fig. 1A and C), whereas siRNA-mediated knockdown of g5Rp reduced both (Fig. 1B and D). Comparative proteomics of g5Rp-overexpressing cells revealed significant alterations in the host proteome (Fig. S1B and C; Dataset S1). Gene Ontology (GO) enrichment analysis identified significant changes in translation factor activity, transmembrane transport, and antioxidant activity (Fig. 1E). KEGG pathway analysis linked g5Rp to autophagy, lysosomal function, ferroptosis, and apoptosis regulation (Fig. 1F). Collectively, these findings indicate that g5Rp promotes ASFV replication by remodeling multiple host cellular pathways.

### g5Rp interacted with eIF5A and RPS15 to promote ASFV replication

**Fig 2:**
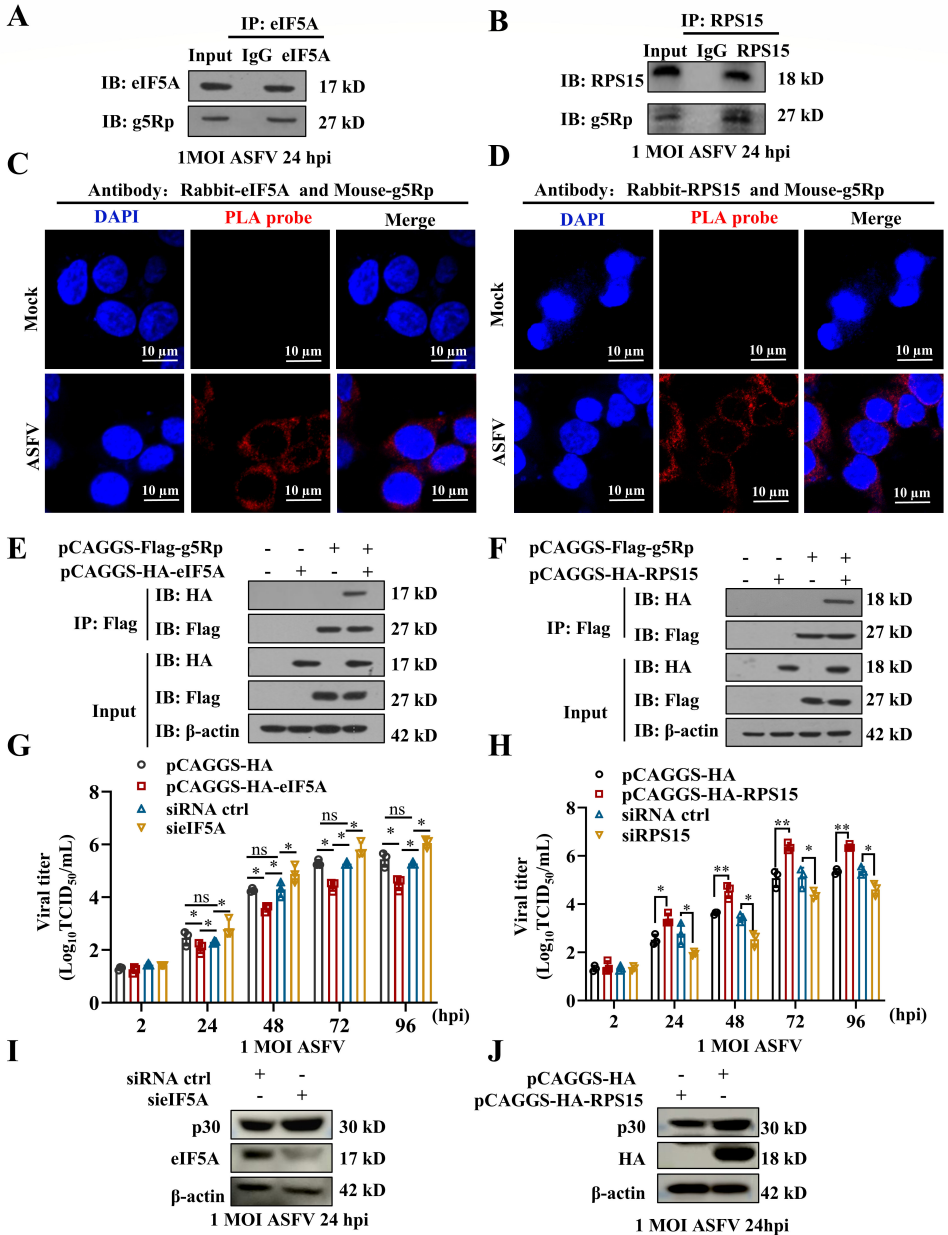
g5Rp interacts with eIF5A and RPS15, modulating host translation to enhance ASFV replication. (**A, B**) Co-IP of ASFV-infected lysates showing g5Rp interaction with eIF5A (**A**) and RPS15 (**B**). ASFV infection at MOI of 1 for 24 hpi. (**C, D**) PLA indicating g5Rp interaction with eIF5A (**C**) and RPS15 (**D**) in ASFV-infected cells. (**E, F**) Validation of g5Rp interaction with eIF5A (**E**) and RPS15 (**F**) by co-transfection in HEK293T cells, immunoprecipitation with anti-Flag, and immunoblotting with anti-HA. (**G, H**) Viral titers measured by TCID50 assay in cells with knockdown or overexpression of eIF5A (**G**) and RPS15 (**H**). (**I, J**) Western blotting analysis showing p30 expression in eIF5A (**I**) knockdown and RPS15 (**J**) overexpression conditions at 24 hpi. β-actin was the loading control. Data are represented as mean ± SD of three independent experiments. Statistical significance was determined using Student’s *t*-test: *, *P* < 0.05; **, *P* < 0.01; ns, not significant.

To elucidate how g5Rp enhances ASFV replication, we identified its interacting proteins via immunoprecipitation-mass spectrometry (IP-MS) (Table S1), focusing on the eukaryotic translation initiation factor 5A (eIF5A) and ribosomal protein S15 (RPS15). These interactions were confirmed under ASFV infection by co-immunoprecipitation (Co-IP) and proximity ligation assay (PLA) (Fig. 2A through D) and were validated in HEK293T cells through co-transfection (Fig. 2E and F). We further found that g5Rp significantly downregulated eIF5A protein levels and inhibited its hypusination, thereby compromising eIF5A’s antiviral restriction activity (Fig. S2A). Functionally, eIF5A knockdown enhanced viral replication and p30 expression, while its overexpression suppressed both (Fig. 2G and I; Fig. S2B). Although g5Rp did not alter total RPS15 levels, it significantly reduced the association of RPS15 with the 40S ribosomal subunit (Fig. S2C through E), indicating impaired ribosomal localization and function. Consistently, RPS15 overexpression further enhanced viral replication (Fig. 2H and J; Fig. S2F). Thus, g5Rp remodeled the host translational machinery by (i) inhibiting eIF5A-mediated antiviral restriction and (ii) disrupting RPS15 ribosomal localization and function, thereby preferentially promoting viral protein synthesis and enhancing ASFV replication efficiency.

### g5Rp disrupted the interaction between eIF5A and RPS15 and inhibited host translation

**Fig 3:**
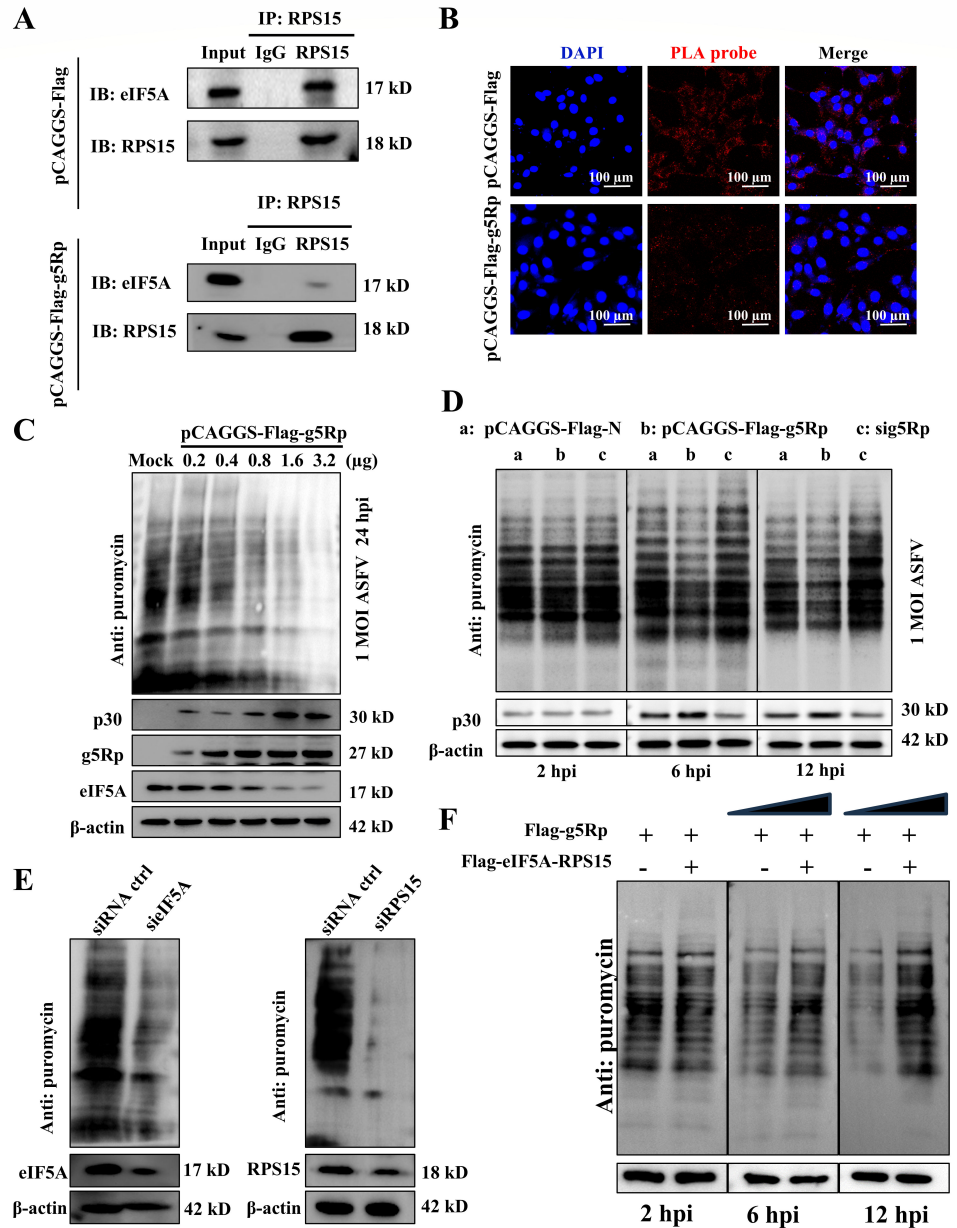
g5Rp disrupts eIF5A-RPS15 interaction and inhibits protein synthesis. (**A**) g5Rp disrupts eIF5A and RPS15 binding. Co-IP using anti-RPS15 antibody on cells transfected with vector (Flag) or Flag-g5Rp, followed by Western blotting analysis. (**B**) g5Rp disrupts eIF5A–RPS15 proximity. PLA signals (red) indicate interactions; nuclei were counterstained with DAPI (blue). (**C**) g5Rp reduces global translation while increasing ASFV p30 protein levels, as assessed by puromycin labeling. (**D**) Time-course study of protein synthesis in ASFV-infected cells (MOI 1) with mock, Flag-g5Rp, or sig5Rp. Western blotting analysis at indicated times post-infection (hpi). (**E**) Knockdown of eIF5A or RPS15 reduces protein synthesis. Cells transfected with control or gene-specific siRNAs were analyzed by puromycin labeling and Western blotting. (**F**) eIF5A–RPS15 fusion protein restores translation. Cells co-transfected with Flag-g5Rp and Flag-eIF5A–RPS15 fusion construct were infected with ASFV and analyzed by Western blotting at indicated hours post-infection (hpi).

eIF5A and ribosome binding are the basis of host translation (25). Therefore, we examined whether g5Rp inhibits host translation by directly disrupting the eIF5A–RPS15 complex. Using Co-IP and PLA, we demonstrated that g5Rp disrupts eIF5A–RPS15 interaction (Fig. 3A and B). In host cells, g5Rp overexpression exhibited dose-dependent and time-dependent suppression of global protein synthesis, as quantified by the puromycin incorporation assay (Fig. 3C and D; Fig. S3A and B). Concurrently, ASFV p30 protein accumulation correlated with increased viral genome replication (Fig. 3C and D; Fig. S3C through E). Furthermore, eIF5A or RPS15 knockdown phenocopied this translational inhibition and induced cell cycle arrest (Fig. 3E; Fig. S3F). Conversely, expression of an eIF5A–RPS15 fusion protein that maintains complex integrity fully restored translation (Fig. 3F). These findings establish the disruption of the eIF5A–RPS15 complex as a key mechanism for g5Rp-mediated enhancement of ASFV replication.

### g5Rp impaired autophagic flux by disrupting the eIF5A-TFEB axis

**Fig 4:**
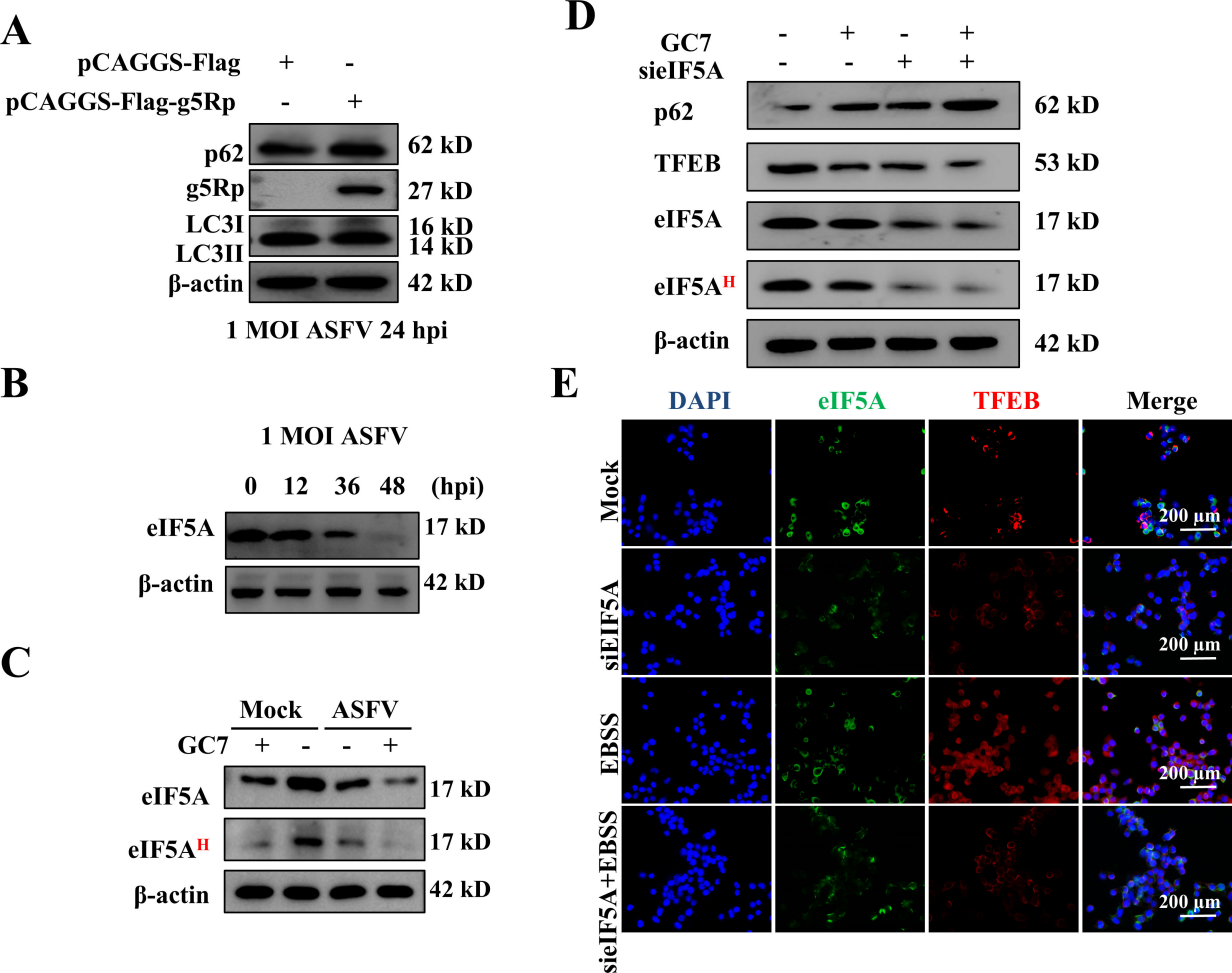
g5Rp impairs autophagic flux by downregulating eIF5A. (**A**) g5Rp overexpression increases p62 accumulation and reduces LC3-II/I ratio in ASFV-infected cells (24 hpi, MOI = 1). (**B**) Time-dependent decrease of eIF5A protein during ASFV infection. (**C**) eIF5A hypusination inhibitor GC7 reduces hypusinated eIF5A in uninfected and ASFV-infected cells. (**D**) eIF5A knockdown or GC7 treatment phenocopies g5Rp effects: elevated p62 with reduced TFEB and total eIF5A. (**E**) eIF5A depletion blocks TFEB nuclear translocation under starvation (EBSS treatment).

We first determined that g5Rp overexpression significantly increased p62 accumulation and decreased the LC3-II/I ratio (Fig. 4A; Fig. S4A), indicating impaired autophagic flux. During ASFV infection, eIF5A protein levels progressively declined (Fig. 4B; Fig. S4B). The eIF5A inhibitor GC7 phenocopied these changes and reduced eIF5A hypusination (Fig. 4C; Fig. S4C). Impaired eIF5A function directly caused autophagic dysregulation, as eIF5A knockdown or GC7 treatment phenocopied g5Rp overexpression, resulting in p62 accumulation and reduced autophagosome formation (Fig. 4D; Fig. S4D and E). Immunofluorescence assay confirmed that eIF5A knockdown blocked starvation-induced TFEB nuclear translocation (Fig. 4E). g5Rp downregulated eIF5A expression and hypusination, thereby disrupting TFEB-mediated autophagy and impairing autophagic flux.

### Identification of key interaction sites in g5Rp for eIF5A or RPS15 binding

**Fig 5:**
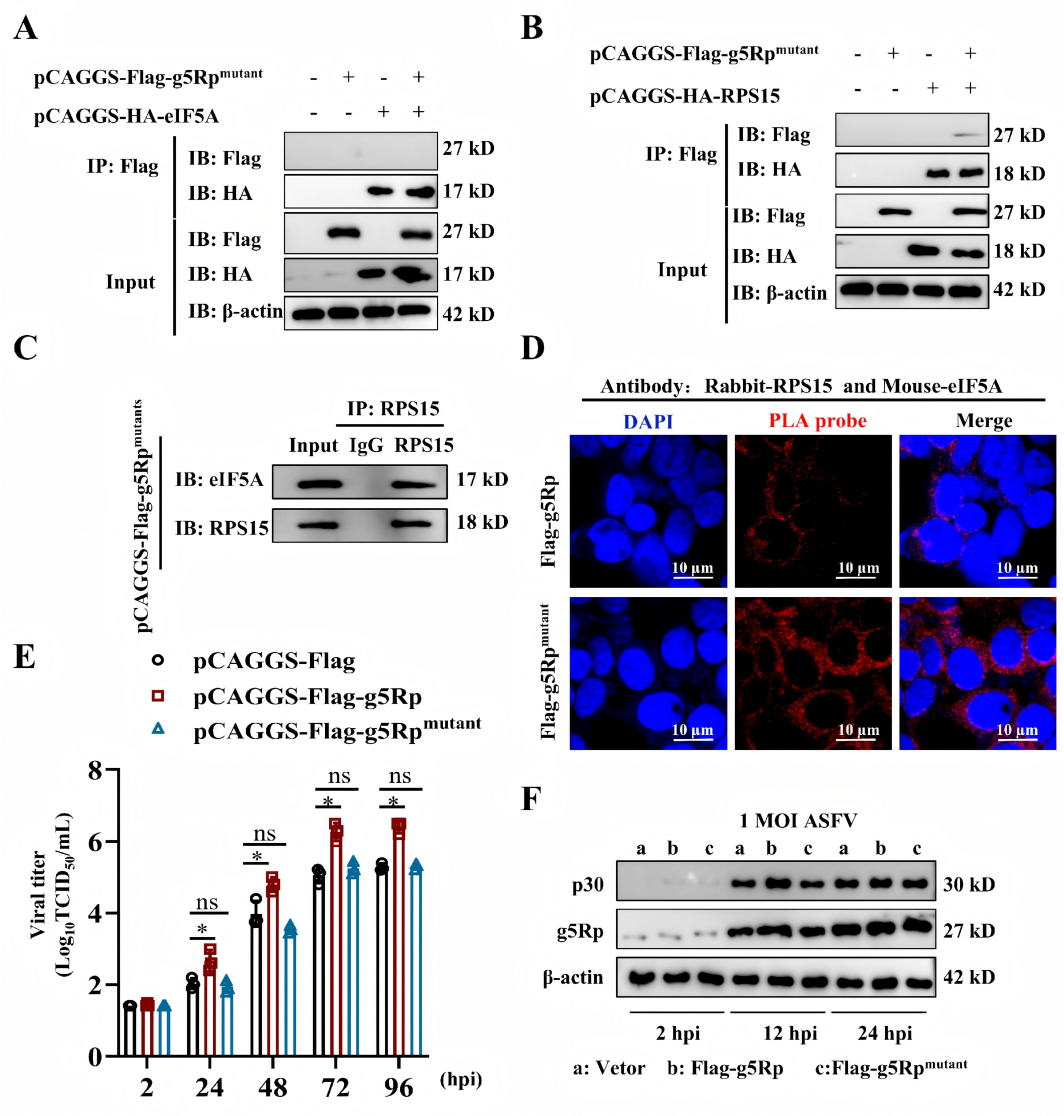
Impact of g5Rp mutant on ASFV replication. (**A, B**) Co-IP analysis shows no interaction between the g5Rp mutant and eIF5A or RPS15. (**C, D**) Co-IP and PLA assays demonstrate restored interactions between eIF5A and RPS15 in the g5Rp mutant. (**E**) TCID_50_ assay evaluates the effect of the g5Rp mutant on ASFV replication. (**F**) Western blotting analysis reveals altered p30 protein expression in the g5Rp mutant. Data are represented as mean ± SD of three independent experiments. Statistical significance was determined using Student’s *t*-test: *, *P* < 0.05; **, *P* < 0.01; ns, not significant.

To identify the specific amino acid binding sites of the three, we determined that g5Rp forms a stable, symmetric homodimer (Fig. S5A and B; Supplementary Material 2Table S2), which not only supports catalytic activity but also serves as a platform for protein interaction. Molecular docking revealed a hydrogen bond–mediated ternary network centered on g5Rp residues SER¹¹⁸, SER²⁰⁶, and ASN⁶¹: eIF5A–ASN⁸³ and RPS15–ARG⁸¹ competed for g5Rp–SER¹¹⁸, while eIF5A–ARG²⁶ and RPS15–ARG⁴⁴ competed for g5Rp-ASN⁶¹ (Fig. S5C through F). Functional analysis showed that alanine (ALA) substitution at SER¹¹⁸, SER²⁰⁶, and ASN⁶¹ (g5Rp mutant) abolished g5Rp–eIF5A binding and attenuated g5Rp–RPS15 interaction (Fig. 5A and B). This restored physiological eIF5A–RPS15 binding (Fig. 5C and D), confirming these residues as critical interaction sites. Consequently, g5Rp lost its wild-type viral promotion capacity (Fig. 5E and F; Fig. S5G and H), indicating that g5Rp’s proviral activity requires an intact eIF5A or RPS15-binding interface.

### 9″-methyl salvianolate B binds ASFV g5Rp and modulates eIF5A and RPS15 interaction

**Fig 6:**
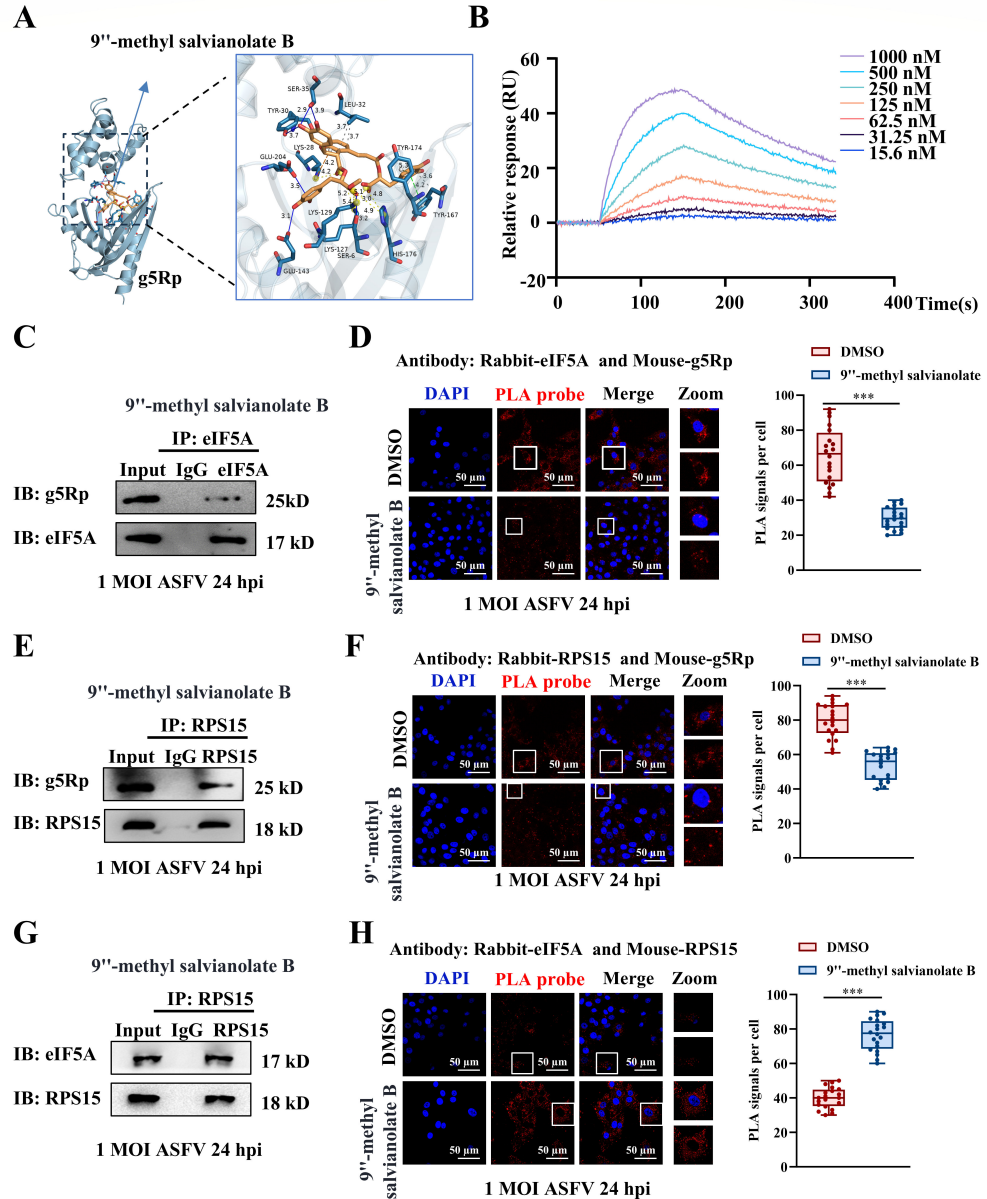
9″-methyl salvianolate B attenuates the interaction between g5Rp and eIF5A or RPS15. (**A**) Molecular docking of 9″-methyl salvianolate B with g5Rp. (**B**) Binding kinetics were analyzed by surface plasmon resonance (SPR). (**C–F**) Co-IP and PLA assays showed that 9"-methylsalvianolate B inhibited the interaction of g5Rp with eIF5A and RPS15. (**G–H**) Co-IP and PLA assays showed that 9"-methylsalvianolate B restored the interaction between eIF5A and RPS15.

Based on the binding site identified by molecular docking, virtual screening identified 9″-methyl salvianolate B as a high-affinity g5Rp inhibitor (Table S3). Molecular docking demonstrated its stable binding to the hydrophobic pocket of g5Rp (Fig. 6A), and surface plasmon resonance (SPR) confirmed strong binding kinetics (Fig. 6B; Fig. S6A and B). The CC50 in 3D4/21 cells was 16.22 µM, with viability exceeding 80% at 10 µM, indicating optimal conditions for functional studies (Fig. S6C and D). Co-IP and PLA showed that g5Rp binding to eIF5A or RPS15 was reduced (Fig. 6C through F), leading to restored eIF5A and RPS15 interactions (Fig. 6G and H).

### 9″-methyl salvianolate B restored host function and inhibited ASFV replication

**Fig 7:**
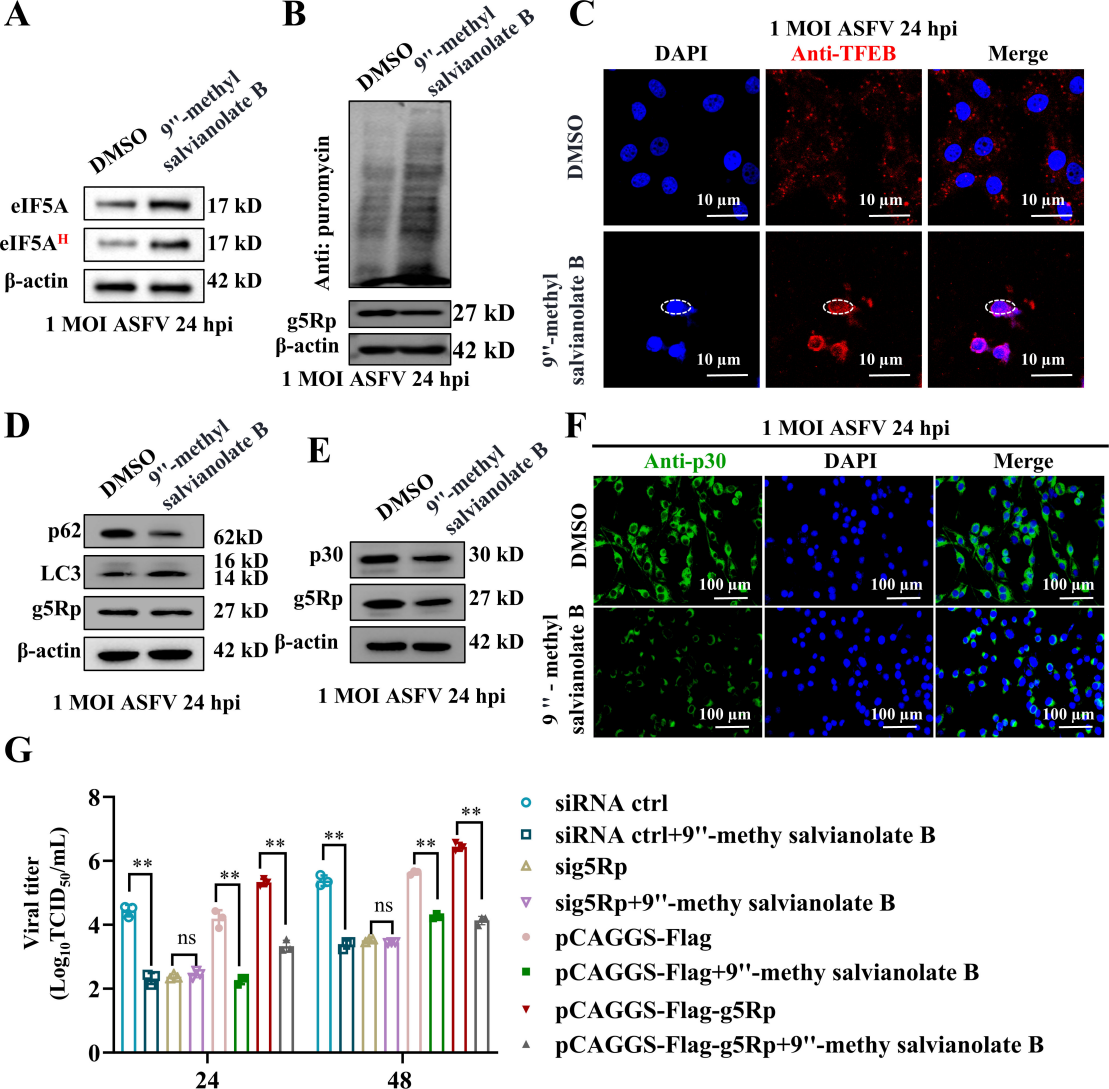
9″-methyl salvianolate B restores host functions and inhibits ASFV replication. (**A**) Western blotting analysis of 9″-methyl salvianolate B effect on eIF5A hypusination. (**B**) Western blotting analysis demonstrating restoration of host protein synthesis by 9″-methyl salvianolate B. (**C**) IFA analysis showing that 9″-methyl salvianolate B promotes TFEB nuclear translocation. (**D**) Western blotting analysis evaluating the impact of 9″-methyl salvianolate B on p62 and LC3 expression. (**E, F**) Western blotting and IFA analyses of 9″-methyl salvianolate B effects on p30 expression. (**G**) TCID_50_ assay evaluating the impact of 9″-methyl salvianolate B on ASFV viral load. Data are represented as mean ± SD of three independent experiments. Statistical significance was determined using Student’s *t*-test: *, *P* < 0.05; **, *P* < 0.01; ns, not significant.

To verify whether 9″-methyl salvianolate B has the function of restoring host translation and autophagy, we first verified its effect on eIF5A hypusination, and the results showed that 9″-methyl salvianolate B could restore eIF5A hypusination (Fig. 7A; Fig. S7A). This compound partially restored host cell protein synthesis (Fig. 7B) and alleviated cell cycle arrest at the G0/G1 phase (Fig. S7B and C). Critically, it promoted TFEB nuclear translocation (Fig. 7C; Fig. S7D), leading to increased LC3-II/LC3-I ratios, reduced p62 levels (Fig. 7D; Fig. S7E), and elevated lysosomal biogenesis (Fig. S7F), which indicates restored autophagic flux. In ASFV-infected cells, 9″-methyl salvianolate B reduced viral p30 protein expression (Fig. 7E and F; Fig. S7G) and suppressed viral replication, even upon g5Rp overexpression (Fig. 7G). These findings establish 9″-methyl salvianolate B as a candidate therapeutic that targets g5Rp to restore eIF5A and RPS15 interactions, reactivate host protein synthesis, and reactivate the autophagy pathway to inhibit ASFV replication.

## DISCUSSION

**Fig 8:**
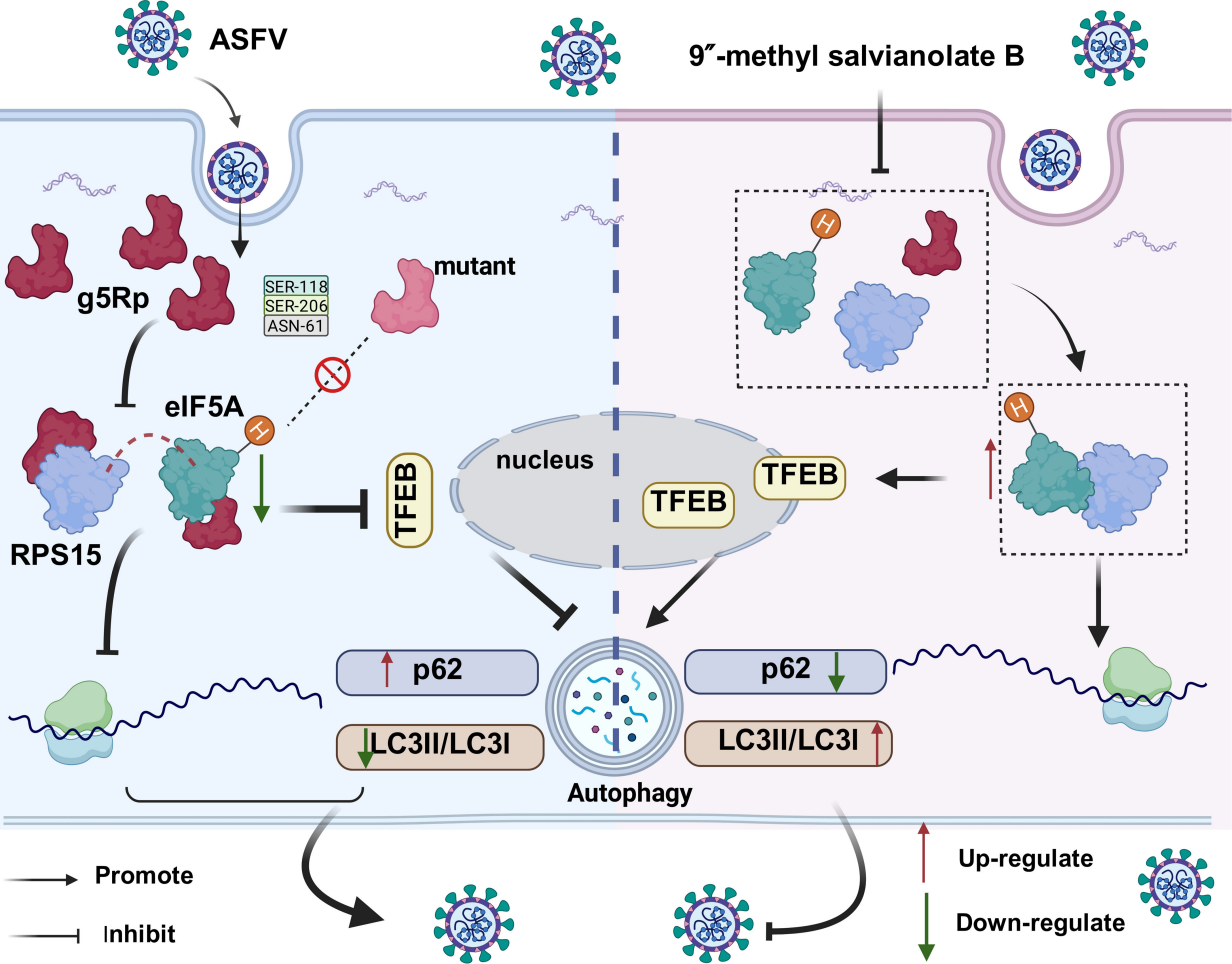
Schematic diagram of the mechanism of g5Rp in ASFV replication.

ASFV causes a highly contagious hemorrhagic disease, incurring catastrophic economic losses globally due to the absence of vaccines or antivirals (26). This therapeutic gap reflects persistent knowledge deficits regarding viral pathogenesis, specifically how ASFV subverts host machinery. Here, we identify the viral protein g5Rp as a key virulence factor that hijacks host translation and autophagy. This dual mechanism advances our understanding of ASFV pathogenesis and nominates g5Rp as a therapeutic target (Fig. 8).

The integration of proteomics and virology has advanced our understanding of viral replication, host antiviral responses, and viral subversion mechanisms (27). In ASFV research, proteomics revealed virus-host interaction dynamics. Infection significantly remodels the host proteome, disrupting biological processes and signaling pathways (28, 29). In this study, proteomics screened the key host factors eIF5A and RPS15 that interact with g5Rp and synergistically regulated host cell translation and autophagy, which provided a theoretical basis for the design of antiviral targets to elucidate a new strategy for efficient replication of ASFV g5Rp by regulating eIF5A and RPS15.

Translation factor-ribosome interactions (e.g., eIF5A binds via its N- and C-terminal domains to address ribosomal arrest and promote viral IRES activity) have been recognized as central to protein homeostasis (30–33). Our study uncovered that g5Rp acts as a viral disruptor by competitively binding both eIF5A and RPS15. This dismantled their functional complex, impairing ribosome assembly, suppressing translation, and inducing G0/G1 arrest—effects that synergize with g5Rp-mediated autophagy dysregulation to drive ASFV-induced proteostasis collapse. This mechanism, distinct from prior reports on isolated translation factors (34, 35) or ribosomal proteins (36–38), positioned g5Rp as a master coordinator of host translational hijacking, offering a molecular lens through which to reinterpret multifactor-ribosome states in viral pathogenesis.

eIF5A maintains autophagic flux by facilitating the translation of autophagy regulators containing polyproline motifs, such as TFEB (39, 40). TFEB serves as the core effector, whose overexpression reverses autophagy impairment caused by eIF5A inhibition, although this regulatory axis exhibits significant context dependency (39, 41). Hypusinated eIF5A participates broadly in viral replication. It functions not only as a critical regulator of Ebola virus gene expression (involving polyamine-dependent mechanisms specific to hypusinated eIF5A) (42), but also contributes to the replication of pathogens, including Marburg virus (MARV) and HIV (41). We therefore investigated whether g5Rp exploits this post-translational modification. Critically, g5Rp downregulated both total and hypusinated eIF5A, inhibiting protein synthesis and autophagy to facilitate ASFV replication. This aligns with established reports that hypusination promotes replication of Kaposi’s sarcoma-associated herpesvirus (KSHV) and Coxsackievirus (32, 43). Although ASFV’s impact on polyamine metabolism requires further investigation, g5Rp-mediated eIF5A reduction sufficiently suppresses autophagy independently of canonical pathways.

Current ASFV inhibitor development focuses on essential enzymes, including the pS273R cysteine protease and dUTPase, providing key foundations for antiviral drug discovery (44–46).

Here, through structure-guided virtual screening coupled with molecular dynamics simulations (47), we identify 9″-methyl salvianolate B—a phenolic compound from Radix Salvia miltiorrhizae with established anti-inflammatory properties (48, 49)—as the first g5Rp protease-specific inhibitor. Mechanistically, it formed stable hydrogen-bonding networks to suppress enzymatic activity, significantly reducing ASFV replication efficacy. This work validated g5Rp as a novel druggable target and delivered a key candidate molecule for anti-ASFV lead optimization.

While this study established g5Rp’s role *in vitro*, several questions remain. First, the *in vivo* efficacy of 9″-methyl salvianolate B requires validation in porcine models, particularly regarding pharmacokinetic optimization for tissue-specific delivery. Second, the interplay between g5Rp and other ASFV immune evasion proteins warrants further investigation, as synergistic interactions may amplify viral pathogenicity. Third, our structural data do not fully explain how g5Rp binding to RPS15 modulates ribosome function, a question addressed by cryo-EM studies of g5Rp-ribosome complexes.

In conclusion, we proposed a model wherein g5Rp acts as a central hub coordinating host translational shutdown and autophagy inhibition to promote ASFV replication. The integration of proteomic, structural, and pharmacological approaches in this study bridges the critical gap between ASFV biology and therapeutic development. Our study not only redefines g5Rp as a multifunctional virulence factor but also pioneers a host-directed antiviral strategy with broad implications for combating complex DNA viruses.

## MATERIALS AND METHODS

### Cells and virus

Porcine alveolar macrophage-derived 3D4/21 cells (ATCC CRL-2843) and HEK293T cells (ATCC 73451) were maintained in DMEM supplemented with 10% FBS, 100 U/mL penicillin, and 100 µg/mL streptomycin at 37°C with 5% CO₂. The ASFV strain China/LN2018/1 was propagated in 3D4/21 cells and stored at −80°C. Viral titers were determined by TCID₅₀. All ASFV-related experiments were performed in a BSL-3 facility.

### Co-immunoprecipitation (Co-IP)

3D4/21 cells were transfected with plasmids and infected with ASFV (MOI = 1, 24 h). Cells were collected and lysed in lysis buffer (50 mM Tris-HCl, pH 7.4, 150 mM NaCl, 5 mM MgCl₂, 1 mM EDTA, 1% Triton X-100, and 10% glycerol) containing 1 mM phenylmethylsulfonyl fluoride (PMSF) and 1× protease inhibitor cocktail (Roche, Basel, Switzerland). Cell lysates were centrifuged (12,000 × *g*, 10 min), and supernatants were pre-cleared by incubation with protein A/G agarose (Sigma) at 4°C for 1 h. The pre-cleared supernatants were then incubated with anti-Flag (M2) agarose beads or with protein G plus agarose beads (MedChemExpress) pre-bound with 1 µg of control IgG antibody overnight at 4°C on a roller. The immunoprecipitation complexes were captured by the beads and subsequently washed five times with the cell lysis buffer. Captured complexes were then analyzed by immunoblotting.

### LC-MS/MS analysis

LC-MS/MS was conducted on a Q Exactive HF-X system (Thermo Fisher) as described (Thermo Fisher Scientific) (50). Briefly, IP-precipitated proteins were separated by SDS-PAGE. Gel bands were treated with reduction/alkylation buffer (10 mM TCEP, 60 mM iodoacetamide, 50 mM NH₄HCO₃) and digested with trypsin (2 µg in 50 mM NH₄HCO₃), and peptides were desalted using C18 tips (Pierce). Samples were lyophilized, dissolved in 0.1% formic acid, and analyzed by LC-MS/MS. Data were processed via Proteome Discoverer 2.2 (FDR <1%, ≥2 unique peptides, fold change ≥2 vs IgG, *P* < 0.05).

### Proximity ligation assay (PLA)

PLA was performed according to published protocols (51). ASFV-infected cells were fixed (4% PFA, 20 min), permeabilized (0.01% Tween-20/PBS, 15 min), and incubated with primary antibodies (rabbit anti-RPS15 and mouse anti-g5Rp). After adding oligonucleotide-conjugated secondary antibodies, signals were detected with orange fluorescence reagent (Sigma DUO92007). Nuclei were stained with DAPI, and images were acquired by confocal microscopy (Leica TCS SP8).

### X-ray crystallography

X-ray data were collected at beamline BL19U1 (SSRF). The g5Rp structure was solved by molecular replacement and refined iteratively in Phenix (52) and Coot (53).

### Surface plasmon resonance (SPR)

Binding kinetics were assessed on a Biacore 8 (GE Healthcare) (54). g5Rp (13,600 RU) was immobilized on a CM5 chip via amine coupling. 9″-methyl salvianolate B (0.156–10 µM in PBS, ≤0.2% DMSO) was injected at 25°C. The equilibrium dissociation constant (KD = 117 nM) was calculated using Biacore Evaluation Software.

### Statistical analysis

Proteomics data were analyzed using the "ClusterProfiler" R package (55). The SPSS software package (SPSS for Windows v13.0; SPSS Inc., Chicago, IL) was used to perform statistical analysis of the data obtained during the experiment. After normality testing, all data conformed to a normal distribution (Shapiro-Wilk test *P* > 0.05). Differences between the experimental and control groups were analyzed by Student’s *t*-test or one-way ANOVA with Tukey’s test using Prism 10 (GraphPad Software, San Diego, CA). Values are expressed in graph bars or a line graph as the mean ± SD of at least three independent biological replicates. *, *P* < 0.05; **, *P* < 0.01; and ***, *P* < 0.001 were considered statistically significant. ns, no significant difference.

For other methods, such as antibody information, plasmid construction, qPCR, and Western blotting, refer to the supplemental material.

## Data Availability

All relevant data are provided in the article and supplemental material. Data related to MS and proteomic analysis of g5Rp were deposited in the ProteomeXChange Consortium via the PRIDE (57) partner repository, with the data set identifier PXD046133 (http://www.ebi.ac.uk/pride). The final coordinates of the g5Rp structure were deposited in the PDB under ID 8WQQ.

## SUPPLEMENTAL MATERIAL

Data Set S1 jvi.01252-25-s0001.xlsx Differentially expressed proteins in response to g5Rp overexpression.

Supplemental figures jvi.01252-25-s0002.docx Figures S1 to S7.

Supplemental tables jvi.01252-25-s0003.docx Tables S1 to S5.

Supplemental text jvi.01252-25-s0004.docx Supplemental methods.

ASM does not own the copyrights to Supplemental Material that may be linked to, or accessed through, an article. The authors have granted ASM a non-exclusive, world-wide license to publish the Supplemental Material files. Please contact the corresponding author directly for reuse.
