# Supplementary material for: Role of g5Rp in African swine fever virus replication: disruption of host translation and autophagy
Source: J Virol. 2025 Dec 15;100(1):e01252-25. doi: 10.1128/jvi.01252-25 (PMC12817904; doi:10.1128/jvi.01252-25)
Supplement: Supplemental figures — Figures S1 to S7. [file jvi.01252-25-s0002.docx]

**Supplementary Material**

**Fig. S1**

**
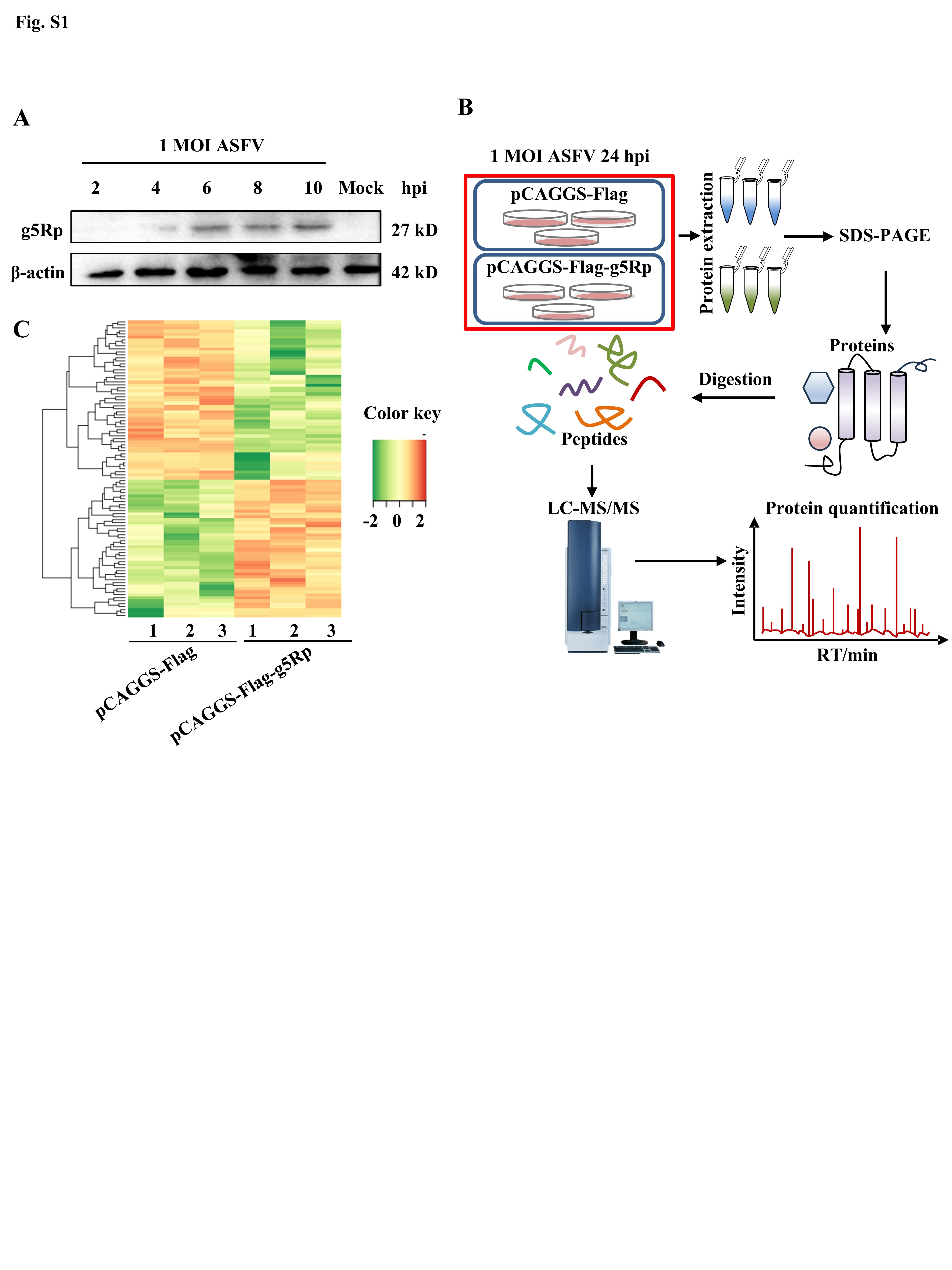
**

**Fig. S1 Identification of differentially expressed proteins in response to g5Rp overexpression during ASFV infection.**

(A) Western blotting analysis of ASFV g5Rp at different time points. (B) Experimental workflow: g5Rp-overexpressing vs control cells subjected to LC-MS/MS proteomics. (C) Heatmap of differentially expressed proteins. Yellow represents upregulated genes, and green represents downregulated genes.

**Fig. S2**

**
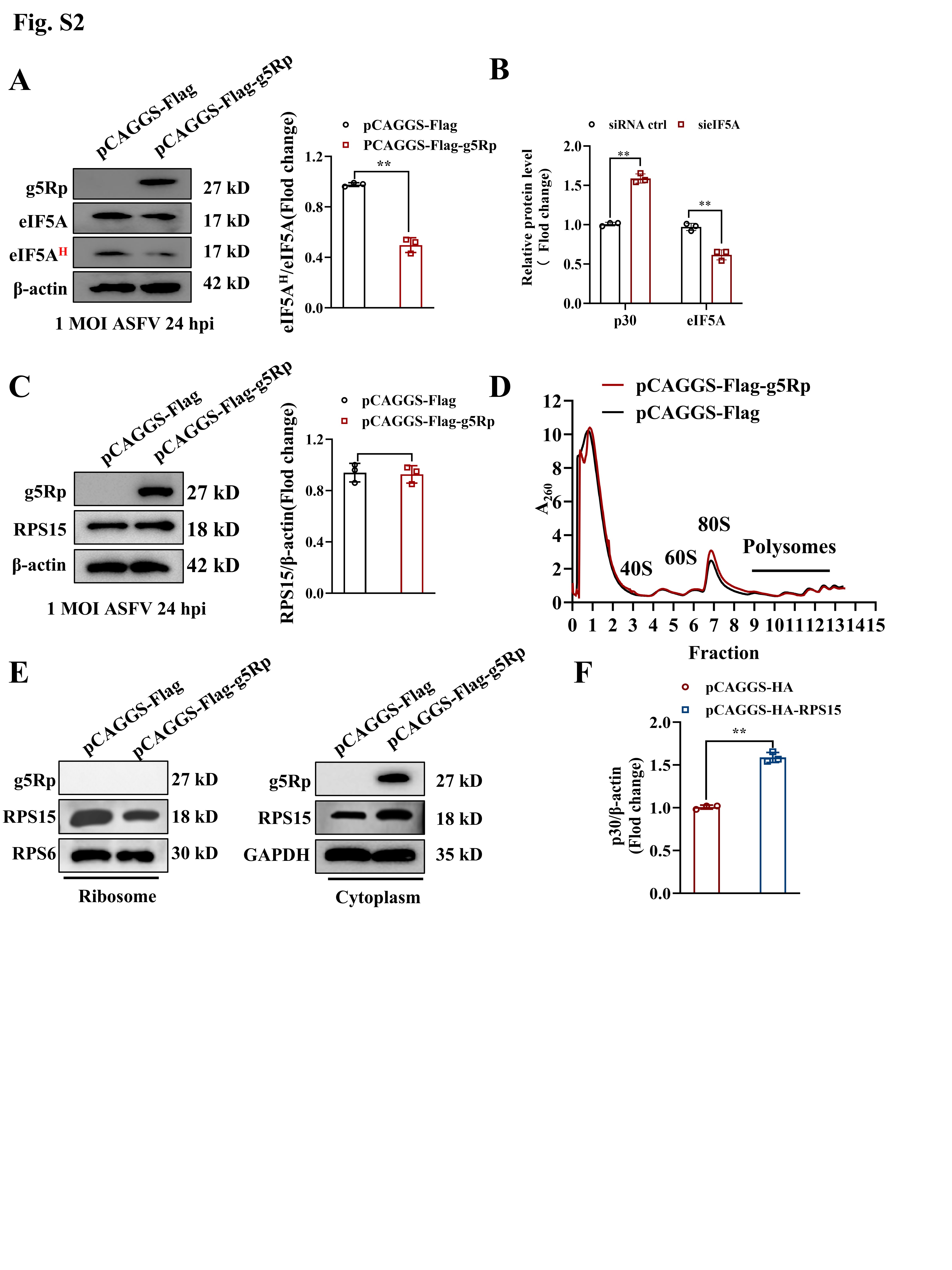
**

**Fig. S2 g5Rp decreases eIF5A and RPS15.** (A) g5Rp downregulates eIF5A and eIF5A^H^. Right panel: Grayscale analysis of eIF5A^H^/eIF5A protein. (B) Analysis of grayscale values of p30 and eIF5A after knockdown of eIF5A in Fig 2I. (C) Western blotting analysis of g5Rp and RPS15 in cells infected with ASFV (1 MOI) for 24 h. Right panel: Analysis of grayscale values of RPS15. (D) Polysome profiling analysis of cells transfected with pCAGGS-Flag or pCAGGS-Flag-g5Rp showing the distribution of ribosomal subunits (40S, 60S, 80S) and polysomes. (E) Western blotting analysis of ribosomal (left) and cytoplasmic (right) fractions showing the levels of g5Rp, RPS15, RPS6 (ribosomal control), and GAPDH (cytoplasmic control). (F) Grayscale analysis for ASFV p30 protein level in RRPS15 overexpression in Fig 2J. Data are presented as mean ± SD from three independent biological replicates. Statistical significance was determined using a two-tailed Student’s t-test. **, *P* < 0.01.

**Fig. S3**

**
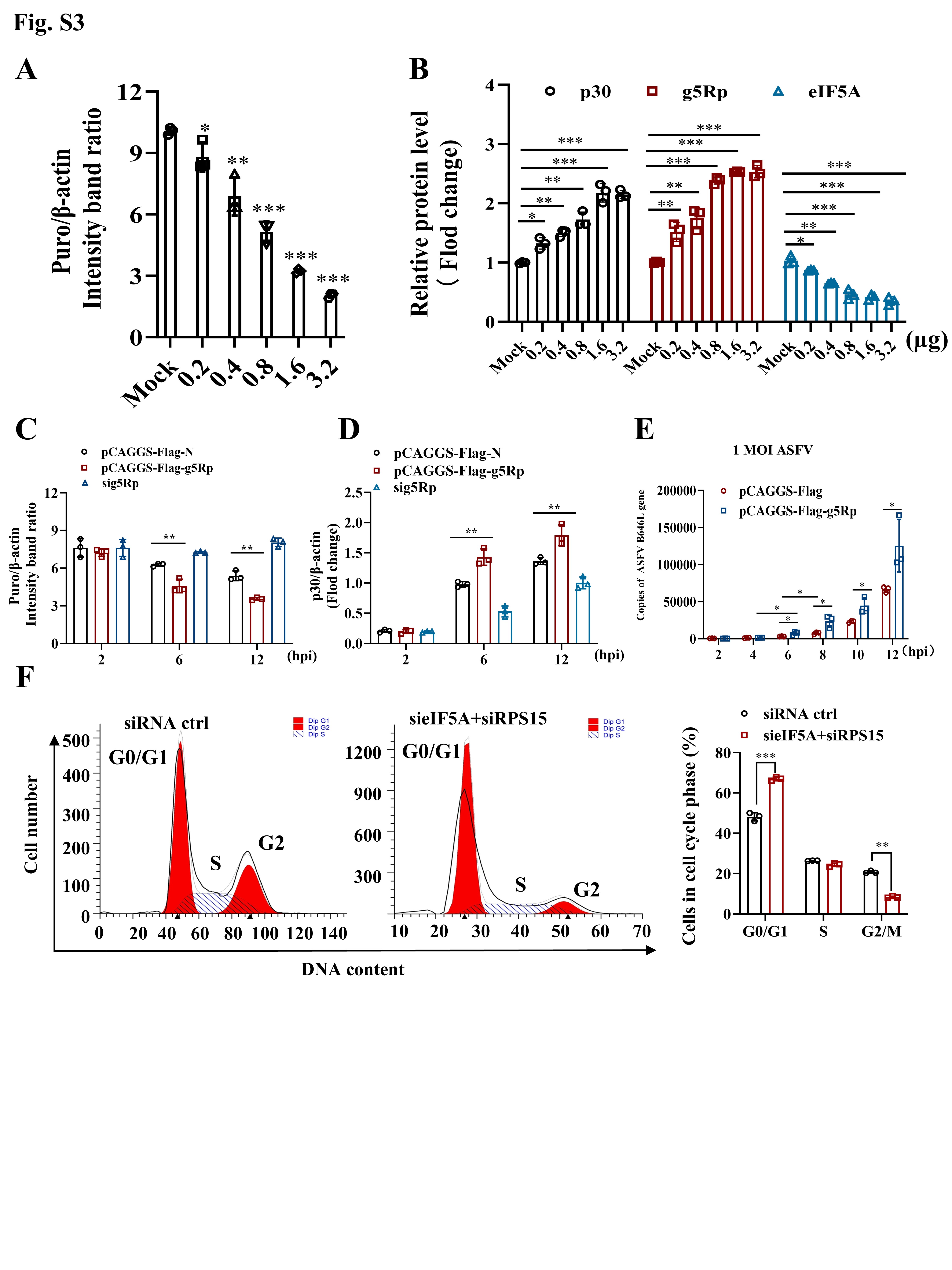
**

**Fig. S3 g5Rp inhibits synthesis of total proteins and induces arrest of cell cycle of 3D4/21 cell line during ASFV infection.** (A) Grayscale analysis of total nascent proteins tagged by puromycin in Fig 3C. (B) Grayscale analysis of p30, g5Rp, and eIF5A in Fig. 3C. (C) Grayscale analysis of total nascent proteins tagged by puromycin in Fig 3D. (D) Grayscale analysis of ASFV p30 in Fig. 3D. (E) Fold change of ASFV genome copies induced by overexpression of g5Rp at various time points. (F) Effect of silencing both eIF5A and RPS15 on host cell cycle. Data are presented as mean ± SD from three independent biological replicates. Statistical significance was determined using a two-tailed Student’s t-test. *, *P* < 0.05; **, *P* < 0.01; ***, *P* < 0.001.

**Fig. S4**

**
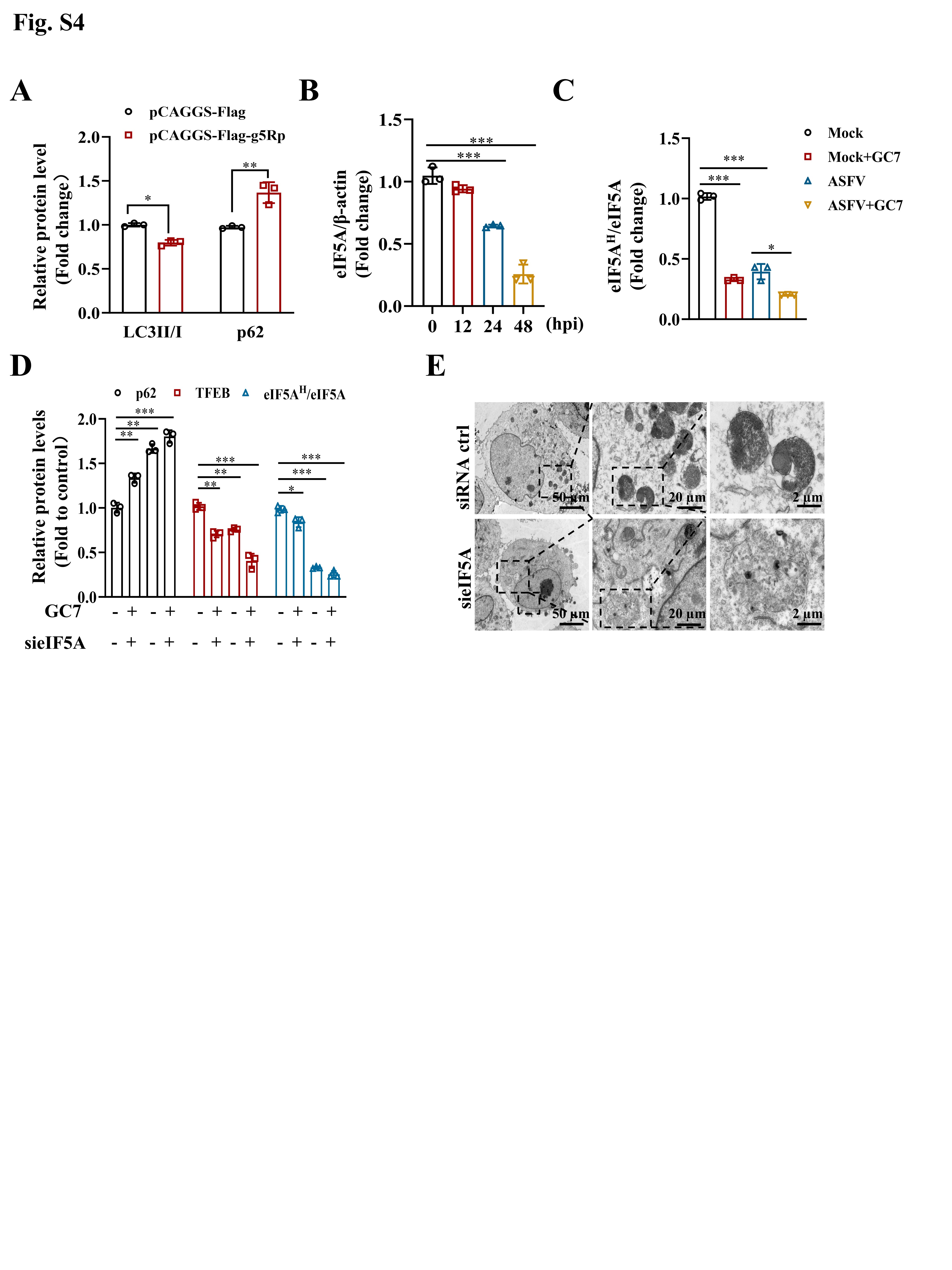
**

**Fig. S4 g5Rp inhibits autophagy by downregulating eIF5A hypusination.** (A) Analysis of grayscale values for LC3II/I and p62 proteins in Fig 4A. (B) Analysis of grayscale values of eIF5A expression in Fig 4B. (C) Analysis of grayscale values for eIF5A and eIF5A^H^ expression in ASFV-infected cells treated with GC7 in Fig 4C. (D) Analysis of grayscale values for p62, TFEB, and eIF5A proteins in GC7 and eIF5A-silenced (sieIF5A) treated groups in Fig. 4D. (E) TEM images of cellular structures in siRNA control (ctrl) and eIF5A-silenced (sieIF5A) cells. Data are presented as mean ± SD from three independent biological replicates. Statistical significance was determined using a two-tailed Student’s t-test. *, *P* < 0.05; **, *P* < 0.01; ***, *P* < 0.001.

**Fig. S5**


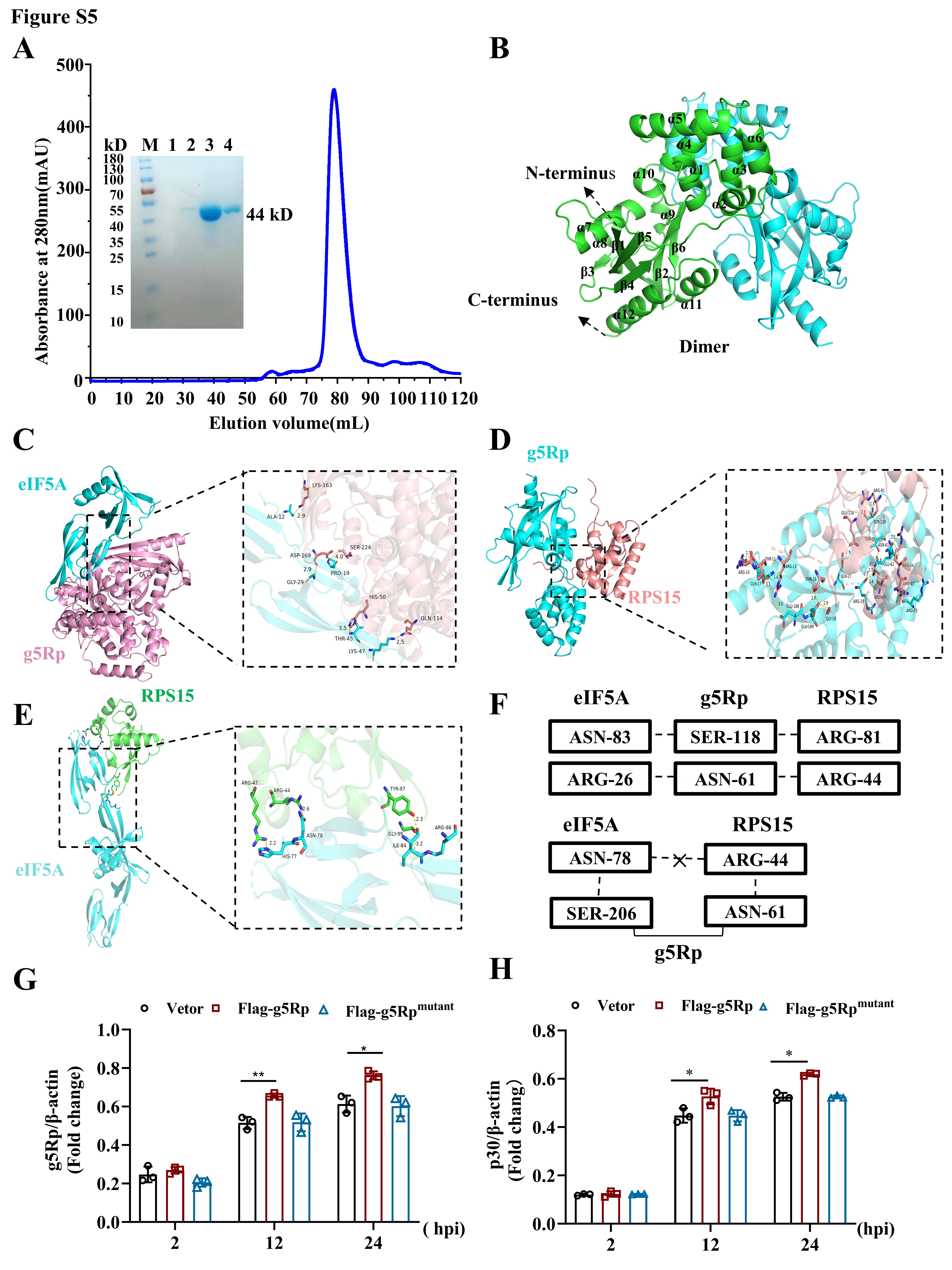


**Fig. S5 Identification of binding sites of g5Rp with eIF5A and RPS15.** (A) Gel filtration profile of protein complex. Inset: SDS-PAGE of protein fractions. Lane M: marker; lanes 1-4: eluted fractions. (B) Cartoon structure of g5Rp dimer. (C-E) Interaction interfaces between eIF5A, g5Rp, and RPS15. Insets show detailed contact residues. (F) Schematic of key interacting residues between eIF5A, g5Rp, and RPS15. (G-H) Analysis of grayscale values for g5Rp and p30 protein expression in Fig 5F_。_Data are presented as mean ± SD from three independent biological replicates. Statistical significance was determined using a two-tailed Student’s t-test. *, *P* <0.05; **, *P* <0.01.

**Fig. S6**

**
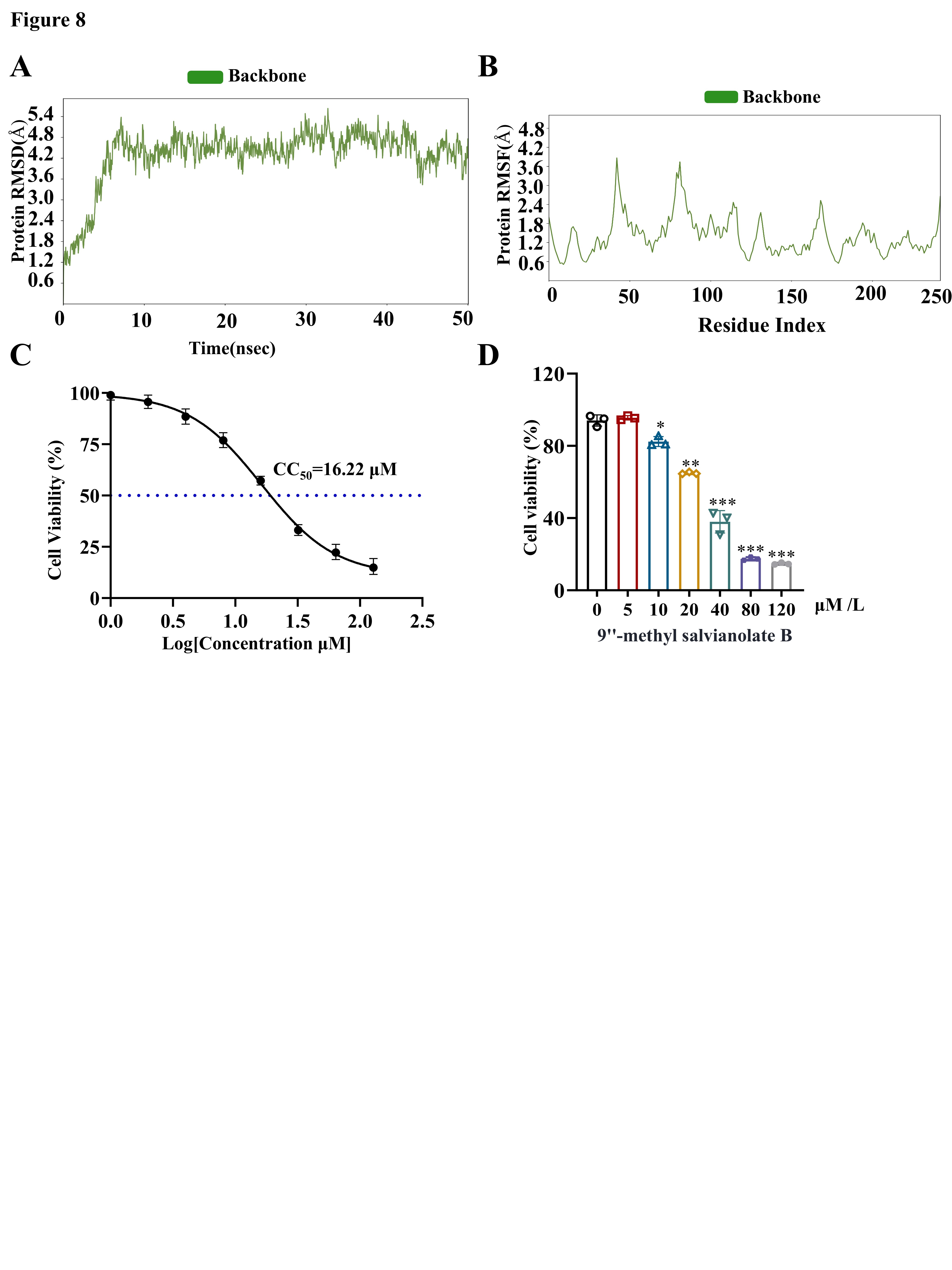
**

**Fig. S6 Comprehensive evaluation of molecular dynamics stability and cytotoxicity/proliferation inhibition.** (A) Residue flexibility (RMSF) during 50 ns molecular dynamics (MD) simulations. (B) Structural stability (RMSD) trajectory throughout MD simulations. Simulations performed in GROMACS v2022.4 using the CHARMM27 force field with periodic boundary conditions. (C) Cytotoxicity in 3D4/21 cells (72 h exposure). CC₅₀ derived from nonlinear regression (GraphPad Prism). (D) Dose-dependent proliferation inhibition (CCK-8 assay). Data are presented as mean ± SD of three independent experiments. Statistical significance was determined using one-way ANOVA with Tukey’s test. *, *P* <0.05; **; *P,* <0.01; ***, *P* < 0.001.

**Fig. S7**


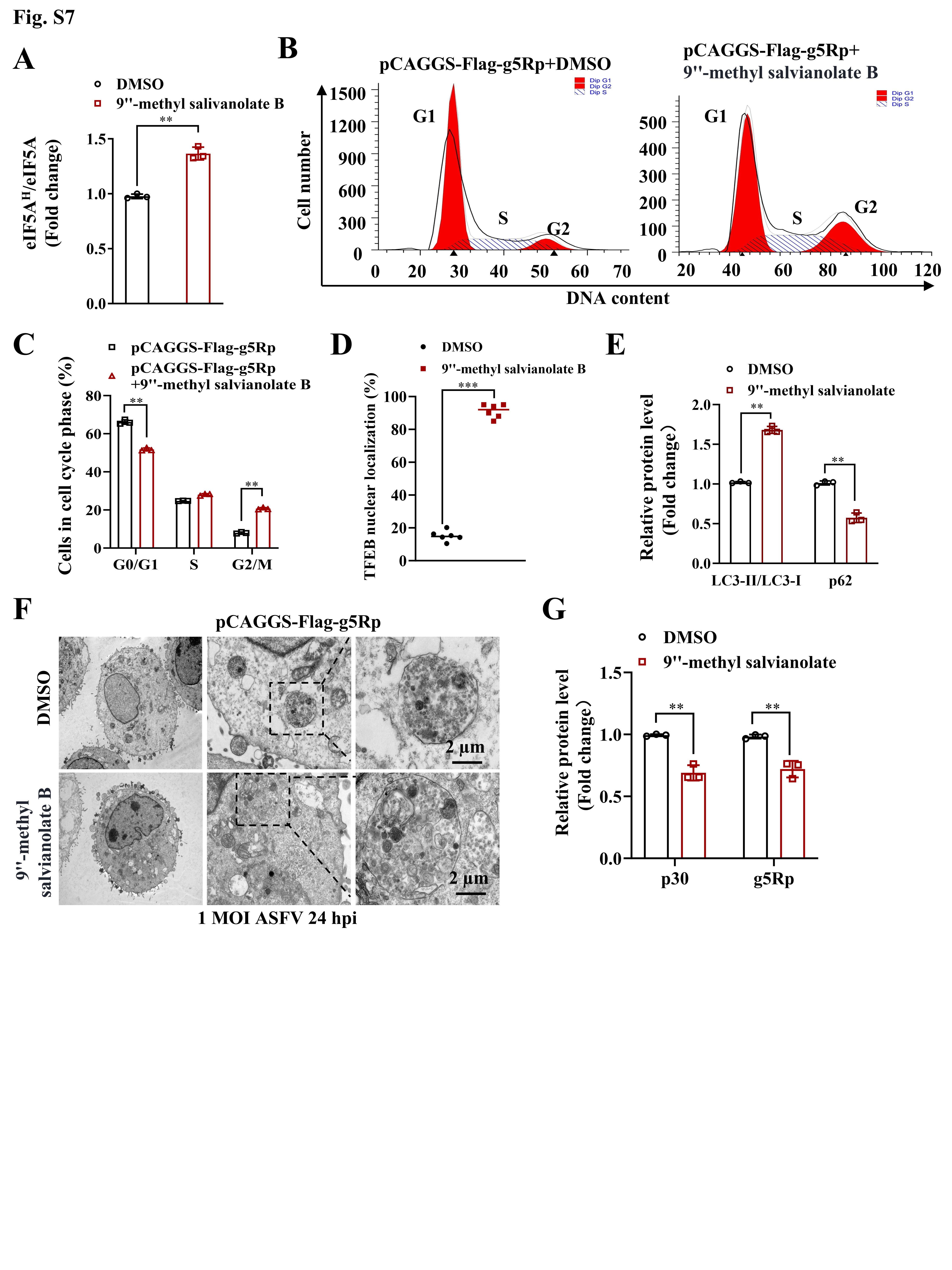


**Fig. S7 The effects of 9″-methyl salvianolate B on ASFV infection.** (A) Grayscale analysis of relative eIF5A^H^ /eIF5A ratio in Fig. 7A. (B-C) Cell cycle analysis of cells treated with pCAGGS-Flag-g5Rp or 9″-methyl salvianolate B. (D) Quantification of relative fluorescence intensity of TFEB in nucleus normalized to DAPI in Fig. 7C. (E) Grayscale analysis of LC3-II/LC3-I and p62 in Fig. 7D. (F) Observation of autophagosomes in response to 9" -methyl salicylate B under transmission electron microscope (TEM). (G) Grayscale analysis of ASFV p30 and g5Rp in Fig. 7E. Statistical significance was determined using a two-tailed Student’s t-test. **, *P* <0.01; ***, *P*<0.001.
