# Supplementary material for: Role of g5Rp in African swine fever virus replication: disruption of host translation and autophagy
Source: J Virol. 2025 Dec 15;100(1):e01252-25. doi: 10.1128/jvi.01252-25 (PMC12817904; doi:10.1128/jvi.01252-25)
Supplement: Supplemental tables — Tables S1 to S5. [file jvi.01252-25-s0003.docx]

**Supplementary Material Table 1-5**

**Table. S1 Proteins interacting with g5Rp identified through immunoprecipitation-mass spectrometry**

| **Accession** | **Description** | **Gene name** | **Sum PEP score** | **Coverage [%]** |
| --- | --- | --- | --- | --- |
| A0A287B356 | Eukaryotic translation initiation factor 5A | *eIF5A* | 23.911 | 31 |
| A0A287AMU5 | 40S ribosomal protein S15 | *RPS15* | 13.395 | 15 |
| A0A287B7Q2 | Sterile alpha motif domain containing 9 | *SAMD9* | 11.57 | 3 |
| F1STC5 | Uncharacterized protein | 9823 | 5.532 | 6 |
| A0A286ZQH3 | Transcription elongation regulator 1 | *TCERG1* | 3 | 1 |
| A0A287A8Q6 | 40S ribosomal protein S27 | *LOC110262272* | 2.388 | 10 |
| A0A287AX39 | 60S ribosomal protein L36a | *RPL36A* | 1.41 | 6 |

**Table. S2 X-ray diffraction data processing and refinement statistics.**

| Parameter | D250R(g5Rp) |
| --- | --- |
| Data collection（resolution） | 2.3Å |
| Space group | P2_1_2_1_2_1_ |
| Unit cell parameters (Å) | 74.20, 77.30, 96.78  90.00, 90.00, 90.00 |
| Resolution range (Å) | 60.40-2.30 (2.42–2.30) |
| Total reflections | 321,887 |
| Unique reflections | 25,470 |
| *R*_merge_ (%)*^b^* | 11.5 (77.8) |
| Avg *I*/σ(*I*) | 15.4 (2.9) |
| Completeness (%) | 100.00 |
| Redundancy | 12.6 (10.8) |
| Refinement |  |
| Resolution (Å) | 30.00–2.30 |
| Number of reflections | 25,174 |
| *R*_work_ (%)*^c^* | 23.75 |
| *R* _free_ (%) | 28.73 |
| R M S Deviations |  |
| Bonds (Å) | 0.034 |
| Angles (°) | 2.045 |
| Average B factor | 37.959 |
| Ramachandran plot quality |  |
| Most favored region (%) | 96.47 |
| Allowed region (%) | 3.53 |
| Disallowed region (%) | 0.00 |

*^a^*Values in parentheses are for the highest-resolution shell.

*^b^R*_merge_ = Σ*_hkl_*Σ*_i_*|*I_i_*(hkl) –〈*I*(hkl)〉|/Σ*_hkl_*Σ*_i_ I_i_*(*hkl*), where *I_i_*(*hkl*) is the observed intensity, and〈*I*(hkl)〉 is the average intensity from multiple measurements.

*^c^R*=Σ*_hkl_*|| *F*_obs_ | – *k* | *F*calc | |Σ*_hkl_*| *F_obs_*|, where *R*_free_ was calculated for a randomly selected 5% of reflections and *R_work_* was calculated for the remaining 95% of reflections used for structure refinement.

**Table S3. Molecular docking scores and MM/GBSA binding energies of the top three hit compounds (kcal·mol^-1^)**

| **TCMSP_ID** | **Chemical compound** | **MM/GBSA** | **Glide-XP** | **Source** | **Chemical structural formula** | |
| --- | --- | --- | --- | --- | --- | --- |
| T4961 | 9''-methyl salvianolate B | -56.32 | -10.408 | Radix *Salvia miltiorrhizae* | | 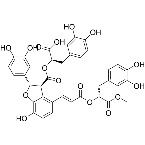 |
| T2951 | β-Cyclodextrin | -48.88 | -13.961 | *Bacillus* | | 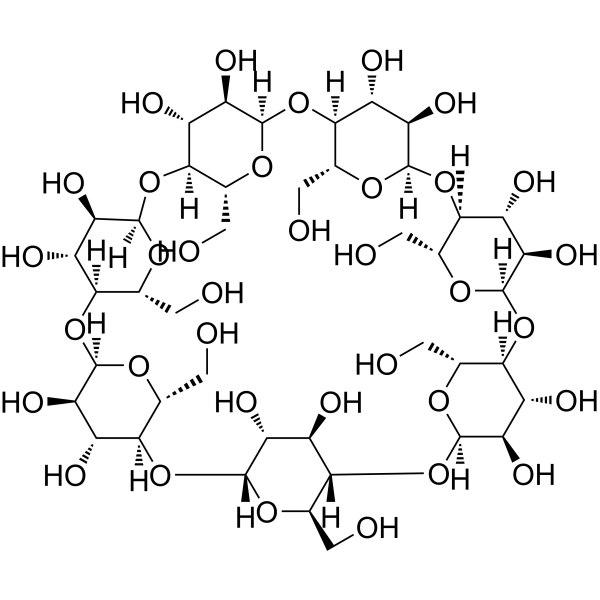 |
| T5S2309 | Hosenkoside K | -43.54 | -10.375 | *Impatiens balsamina* Linn | | 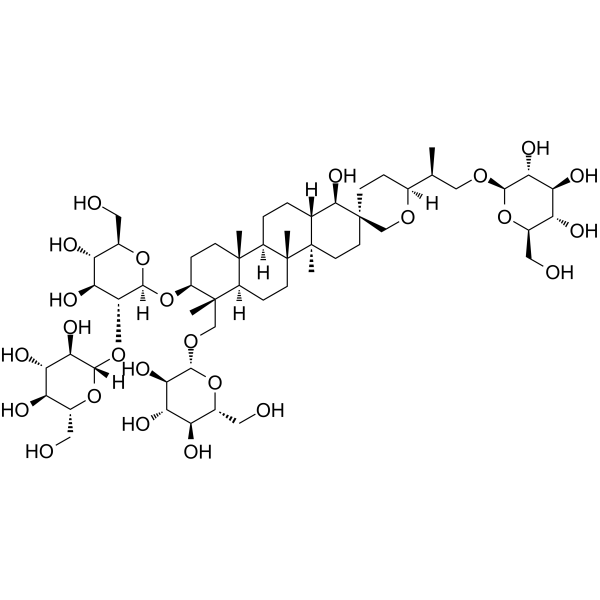 |

MM/GBSA, Molecular Mechanics/Generalized Born Surface Area

**Table. S4**

**Sequences of siRNAs**

| siRNA name | Sequence |
| --- | --- |
| siRNA-ctrl | F: 5′-UUCUCCGAACGUGUCACGUTT-3′  R: 5′-ACGUGACACGUUCGGAGAATT-3′ |
| g5Rp-715 | F: 5′-CAUUAAACCUGGAGCCUAUTT-3′  R: 5′-AUAGGCUCCAGGUUUAAUGTT-3′ |
| g5Rp-384 | F:5′-GGAACACUUCUAUGGGAAATT-3′  R:5′-UUUCCCAUAGAAGUGUUCCTT-3′ |
| g5Rp-409 | F:5′-AGGGUAAGCCGAAGGAAGATT-3′ R:5′-UCUUCCUUCGGCUUACCCUTT-3′ |
| eIF5A-440 | F: 5′- GCAAGGAGAUUGAGCAGAATT-3′  R: 5′- UUCUGCUCAAUCUCCUUGCTT-3′ |

| eIF5A-524 | F: 5′-CAAUCAAGGCCAUGGCAAATT-3′  R: 5′-UUUGCCAUGGCCUUGAUUGTT-3′ |
| --- | --- |
| eIF5A-346 | F: 5′-GGACUUCGAGACAGGAGAUTT-3′  R: 5′-AUCUCCUGUCUCGAAGUCCTT-3′ |
| RPS15-273 | F: 5′- GCAUGGUUGGUGUUUACAA-3′  R: 5′- UUGUAAACACCAACCAUGC-3′ |
| RPS15-310 | F: 5′-GGUUGAAAUCAAACCGGAA-3′  R: 5′-UUCCGGUUUGAUUUCAACC-3′ |
| RPS15-73 | F: 5′-GCUGGAUAUGAGCUACGAA-3′  R: 5′-UUCGUAGCUCAUAUCCAGC-3′ |

**Table S4. List of small interfering RNAs (siRNAs) and their sequences.** This table provides a list of the small interfering RNAs (siRNAs) used in the experiments, along with their corresponding sequences. Each siRNA is composed of a sense strand (F) and an antisense strand (R), used to target and silence specific genes. The table is organized by the siRNA name, with detailed nucleotide sequences provided for each siRNA. siRNA name: The identifier for each siRNA, used to distinguish different siRNAs. For example, "siRNA-ctrl" refers to the control siRNA. Sequence: Corresponding nucleotide sequences for the sense strand (F) and antisense strand (R).

**Table. S5**

**Sequences of primer**

| Gene name | Sequence |  |
| --- | --- | --- |
| *B646L*(p72) | | F: 5′-CACGGTGGAGACATCATCGT-3′  R: 5′-GCTGTTGCTGGTGTTGTTGT-3′ |
| *E183L*(p54) | | F: 5′-GCTACGGTACAGGAGGAACG-3′  R: 5′-CGTGCCAGTTGTTGTTGTAG-3′ |
| *P204L*(p30) | | F: 5′-TACAGCCAGAGCATTAGCAAC-3′  R: 5′-TGTTCCTGCTGTTGTTGGTGT-3′ |
| *D250R*(g5Rp) | | F: 5′-TACGGCTACAGCTGCGATCA-3′ R: 5′-GTCGTTGTTGCTTGCGTTCT-3′ |

**Table. S5 Primer sequences used for quantitative real-time PCR analysis of gene expression.** Each gene's specific forward (F) and reverse (R) primer sequences are provided, detailing the sequences used for amplification in the PCR reactions. These primers were designed to specifically amplify the target gene regions to evaluate their expression levels accurately.
