# Supplementary material for: Role of g5Rp in African swine fever virus replication: disruption of host translation and autophagy
Source: J Virol. 2025 Dec 15;100(1):e01252-25. doi: 10.1128/jvi.01252-25 (PMC12817904; doi:10.1128/jvi.01252-25)
Supplement: Supplemental text — Supplemental methods. [file jvi.01252-25-s0004.docx]

**Supplementary Materials: Materials and Methods**

**Cells, virus, and antibodies**

Porcine alveolar macrophage-derived 3D4/21 cells (ATCC CRL-2843) and Human Embryonic Kidney 293 (HEK293T ATCC CRL-3216) cells were routinely cultured in complete Dulbecco’s Modified Eagle’s medium (DMEM) supplemented with 10% fetal bovine serum (FBS), 100 U/mL penicillin, and 100 µg/mL streptomycin at 37 °C in an incubator with 5% CO_2_. The ASFV strain China/LN2018/1 was propagated in 3D4/21 cells and preserved at -80 °C. Viral titers were determined based on TCID_50_. All experiments related to ASFV were conducted at the BSL-3 laboratory of China Animal Health and Epidemiology Center.

Anti-FLAG rabbit monoclonal antibody (14793) was purchased from Cell Signaling Technology. Anti-FLAG (F1804) and anti-puromycin (ZMS1016) mouse monoclonal antibodies were purchased from Sigma-Aldrich. Anti-HA (HT301-01), anti-HA rabbit monoclonal antibody (AB0025), and anti-β-actin mouse monoclonal antibody (SC-8432) were obtained from TransGen Biotech, Abways Technology, and Santa Cruz Biotechnology, respectively. Anti-p62 rabbit monoclonal antibody ([T55546](http://www.ab-mart.com.cn/page.aspx?node=%2077%20&id=%201786)), Anti-LC3B rabbit monoclonal antibody([T55992](http://www.ab-mart.com.cn/page.aspx?node=%2077%20&id=%202227)), and anti-TFEB rabbit monoclonal antibody (TB4342) were purchased from Abmart. Anti-FLAG (F1804) and anti-puromycin (ZMS1016) mouse monoclonal antibodies were purchased from Sigma-Aldrich. Fluorescein isothiocyanate (FITC)-conjugated goat anti-mouse (BA1101) and DyLight 594-conjugated goat anti-rabbit (BA1142) immunoglobulin G (IgG) antibodies were sourced from Boster. Anti-p30 mouse monoclonal antibody was provided by Professor Changjiang Weng. Horseradish peroxidase-conjugated goat anti-mouse (RK244131) and anti-rabbit (RJ242536) immunoglobulin G (IgG) antibodies were purchased from Thermo Fisher Scientific. Duolink® PLA oligonucleotide-linked anti-mouse IgG (DUO92004) was purchased from Sigma-Aldrich (St. Louis, MO). Anti-eIF5A rabbit monoclonal antibody (D290463），anti-eIF5A mouse monoclonal antibody (D194595), anti-RPS15 rabbit polyclonal antibody (D223778), anti-RPS6 mouse monoclonal antibody (D190813), puromycin (E607054), and GC7 ([A429345](https://store.sangon.com/productDetail?productInfo.code=A429345)) were purchased from Sangon Biotech. Anti-Hypusine rabbit monoclonal antibody (ABS1064) was purchased from Millipore. Anti-g5Rp polyclonal antibody was obtained from a mouse immunized with purified full-length g5Rp expressed using the pET-32a vector in Escherichia coli. Furthermore, 9''-methyl salvianolate B (T4961) was purchased from TargetMol.

**Construction of the recombinant plasmid**

The g5Rp gene was amplified from the ASFV genome (GenBank accession number: OP856591.1) and cloned into the pCAGGS-Flag-N vector (Addgene) to generate the expression plasmid pCAGGS-Flag-g5Rp. Similarly, hemagglutinin (HA)-tagged eIF5A and RPS15 fragments were subcloned into the pCAGGS vector (Addgene) to generate the expression plasmid pCAGGS-HA-eIF5A. pCAGGS-HA-RPS15. The g5Rp mutant and eIF5A and RPS15 fusion expression vectors were synthesized and ligated into the pCAGGS-Flag-N vector by Shanghai Sangon. The g5Rp gene was cloned into pET-32a to obtain a pET-32a-g5Rp prokaryotic expression vector.

**siRNA interference assay**

Target-specific siRNAs were carefully designed and validated to achieve efficient silencing of ASFV g5Rp expression in infected cells, ensuring robust analysis of the functional role of the viral protein. Three siRNAs targeting the g5Rp, eIF5A, and RPS15 transcripts and an siRNA control (negative control) were synthesized by Sangon Biotech (Shanghai, China). siRNAs targeting either g5Rp, eIF5A, and RPS15, or negative control sequences, were transfected into 3D4/21 cells using the transfection reagent Solarbio L3200 following the manufacturer’s recommended procedures. After 6 h, the Roswell Park Memorial Institute 1640 medium supplemented with 10% FBS was replaced and inoculated with ASFV (multiplicity of infection [MOI]=1). Finally, the 3D4/21 cells were subjected to western blotting after 24 h. The Supplemental Table S4 lists the siRNA sequences.

**Analysis of peptide patterns by LC-MS/MS**

LC-MS/MS analysis was performed on a Q Exactive HF-X mass spectrometer coupled to an UltiMate 3000 RSLCnano system (Thermo Fisher Scientific). The experimental procedure was described in a previous study (1). First, the proteins were extracted and enzymatically digested. Total proteins pulled down by immunoprecipitation (IP) were separated using sodium dodecyl sulfate-polyacrylamide gel electrophoresis. The gels containing the protein bands were treated with buffer containing 10 mM Tris (2-carboxyethyl) phosphate, 100% acetonitrile, 60 mM iodoacetamide, and 50 mM NH_4_HCO_3_. Subsequently, the proteins were digested into peptides using a trypsin buffer (2 µg trypsin and 50 mM NH_4_HCO_3_). The resulting peptides were purified using Pierce C18 Tip (87784; Thermo Fisher Scientific). Next, the samples were lyophilized to powder form, dissolved in 100 µL of 0.1% formic acid solution (prepared in sterilized water), treated with a desalting column, and stored at -20 °C for MS, followed by LC-MS/MS data analysis using an ultra-high-resolution combinatorial liquid-mass spectrometer. Raw MS data were processed using Proteome Discoverer software (version 2.2; Thermo Fisher Scientific, USA), and peak generation, precursor mass recalibration, reporter ion intensity extraction, and reporter ion intensity ratio calculations were performed. For each MS/MS spectrum, the top 10 most intense peaks per 100-Da window were extracted for the database search. Amino acid sequences were searched using the UniProt database (UniProt release date: April 10, 2018, search number 328615, http://www.uniprot.org) and Proteome Discoverer software was used to assemble the peptides and screen the identification results. Peptide identification required: false discovery rate (FDR) <1%, minimum 2 unique peptides, and fold-change ≥2 vs. IgG control (Student’s t-test, p<0.05).

**Total RNA extraction and qRT-PCR**

Total RNA was extracted from ASFV-infected 3D4/21 cells using TRIzol reagent and subsequently reverse-transcribed into complementary DNA (cDNA) using M-MLV reverse transcriptase (Promega) according to the manufacturer’s protocol. Quantitative RT-PCR was performed in triplicate using the LightCycler® Nano software (Version 1.0, Roche). Relative mRNA levels were normalized to those of glyceraldehyde 3-phosphate dehydrogenase and analyzed using the 2^-ΔΔCt^ method (2). The Supplemental Table S5 lists the primer sequences.

**Western blotting analysis**

Briefly, 3D4/21 cells were lysed with radioimmunoprecipitation assay (RIPA) lysis buffer containing phenylmethanesulfonyl fluoride (PMSF) for 30 min and centrifuged at 12,000 g for 15 min at 4 °C. The extracted proteins were denatured by boiling in 5× sodium dodecyl sulfate buffer solution for 10 min, followed by electrophoresis. Proteins were transferred to a cellulose nitrate membrane, which was blocked with 5% skim milk powder at 37 °C for 2 h. membranes were incubated with the corresponding antibodies overnight at 4 °C, followed by washing with Tris-buffered saline containing 0.1% Tween 20 (TBST) three times for 10 min each. The membrane was incubated with horseradish peroxidase-labeled diazepam (1:5,000) at room temperature, followed by another wash with TBST, three times for 10 min each. Finally, the membranes were visualized using an electrochemiluminescence imager (Tanon 5200, China).

**Immunoprecipitation (IP)**

3D4/21 cells were transfected with a plasmid and subsequently infected with African swine fever virus (ASFV) at a multiplicity of infection (MOI) of 1 for 24 hours. Following infection, the cells were lysed using IP lysis buffer (Beyotime, China) and gently centrifuged. The resulting supernatant was incubated with 50 µL of protein A/G agarose (Sigma-Aldrich, Saint Louis, USA) at 4 °C for 1 hour, followed by immunoprecipitation (IP) using anti-Flag or anti-IgG antibodies overnight at 4 °C. The protein complexes were then incubated with 60 µL of protein A/G agarose at room temperature for 2 hours and subsequently analyzed by western blotting.

**Protein expression, crystallization, data collection, and structural characterization**

The pET32a-g5Rp plasmid was transformed into BL21 (DE3) cells and induced to express g5Rp using 1-mM IPTG (Isopropyl β-D-1-thiogalactopyranoside) at 18 °C for 16 h. Recombinant proteins were purified to homogeneity using Ni-NTA affinity chromatography under optimized conditions, ensuring high purity for downstream biochemical and structural analyses. Cells expressing His-tagged g5Rp were lysed using a JY92-IIN Ultrasonic Homogenizer (Ningbo Novartis Biotech Co., Ltd.), purified by affinity chromatography on HisTrap HP columns (GE Healthcare, USA) following standard protocols, and pre-balanced with hydrolytic buffers (pH 7.4, 20 mM Tris-HCl, and 500 mM NaCl). Target proteins were washed with elution buffer (pH 7.4, 20 mM Tris-HCl, 500 mM NaCl, and 500 mM imidazole). The eluted fraction was condensed and further purified on a Superdex 200 column (GE Healthcare; 80 mL, 20 mM NaCl, and 200 mM NaCl [pH 7.4]). Optimal crystallization conditions were established for g5Rp through systematic screening of the buffer composition, temperature, and precipitant concentration, yielding high-quality crystals suitable for X-ray diffraction analysis. Finally, the g5Rp was condensed to a concentration of 10 mg/mL for crystallization. Crystallization screening was performed at 16 °C using the static droplet steam-diffusion method. To yield optimal g5Rp crystals, the crystallization conditions were refined as follows: 0.1 M HEPES sodium, pH 7.5, 1.5 M lithium sulfate monohydrate, and 1 µL protein solution (20 mM Tris-HCl and 200 mM NaCl, 8.5 mg/mL; pH 7.4) by vapor diffusion in 1 µL reservoir solution.g5Rp crystals were cryoprotected in reservoir solution supplemented with 25% (v/v) glycerol and flash-cooled in liquid nitrogen. X-ray diffraction data were collected at 100 K on beamline BL19U1 of Shanghai Synchrotron Radiation Facility (SSRF) using a DECTRIS EIGER X 16M detector.

**Electron microscopy assay**

Electron microscopy was employed to investigate the formation of autophagosomes. Cells were embedded in LR White resin, as previously described(3), and sectioned using a Leica UC7 ultramicrotome. Cells were fixed with 2.5% glutaraldehyde and 1% osmium tetroxide, dehydrated through an ethanol series, and embedded in Epon 812 resin. Ultrathin sections (70 nm) were stained with uranyl acetate and lead citrate, then examined using a Tecnai G2 Spirit BioTWIN transmission electron microscope at 80-120 kV to visualize cellular ultrastructure.

**Polysome profiling analysis**

At 6 h post-transfection with either pCAGGS-Flag or pCAGGS-Flag-g5Rp, 3D4/21 cells were infected with ASFV and maintained at 37°C in a 5% CO₂ incubator for 1 h. The infection was allowed to proceed for 24 h. Prior to harvest, cells were treated with 100 μg/mL cycloheximide (Sigma-Aldrich) for 15 min at 37°C to arrest protein synthesis. Cells were then lysed on ice using RIPA buffer (50 mM Tris-HCl, pH 7.4, 150 mM NaCl, 1% NP-40, 0.5% sodium deoxycholate, 0.1% SDS) containing protease inhibitors. After centrifugation at 13,000 × g for 10 min at 4°C, the clarified supernatant was collected. Linear 10-50% (w/v) sucrose density gradients were prepared in SW41 ultracentrifuge tubes (Beckman Coulter) using a Gradient Master (BioComp Instruments). Aliquots (1 mL) of the supernatant were layered onto the gradients and centrifuged at 38,000 × g for 3 h at 4°C in an SW41 rotor (Beckman Coulter). Gradient fractionation was performed using a piston gradient fractionator (Brandel) with continuous monitoring at 254 nm (Bio-Rad). Fractions corresponding to non-ribosomal, 40S-80S, and polysomal components were separately pooled for downstream analysis.

**Virtual screening**

In this study, we employed virtual screening to identify potential inhibitors of the target enzyme, ASFV g5Rp. Virtual screening of small-molecule compounds targeting the g5Rp active site was performed using Schrödinger Suite (Glide-XP scoring and MM/GBSA binding free energy calculations). The coordinates of the crystal structures of PDB: 7DNU and g5Rp were superimposed, and the root mean square deviation (RMSD) was only 0.364 Å, indicating that the two crystal structures were relatively similar. Therefore, we used the crystalline small-molecule ligand in this structure as the reference active pocket and performed structure-based virtual screening of this binding pocket. Based on the pocket site where the small-molecule inhibitor was located, the docking lattice center was set as the crystalline ligand, the docking outer box size was set similar to the crystalline ligand size, and the inner box size was set to 10 Å. Molecular docking was performed using the Schrödinger Virtual Screening Workflow module. The database used for screening was LigPrep-prepared three-dimensional structures. The compounds with the lowest binding energies were selected for further analysis.

**Molecular docking to validate binding activity between g5Rp and eIF5A or RPS15**

Protein-protein docking was performed using HDOCK server (version 2021) with default parameters. The structure of eIF5A was obtained from the RCSB PDB (PDB ID: 3CPF). The RPS15 protein (ID A0A287AMU5) was obtained from the Uniprot Protein Database. PyMOL was used to isolate the original ligands and protein structures, dehydrate them, and remove the organic matter. The LigPlus software (http://www.ebi.ac.uk/thornton-srv/software/LigPlus/) was used to analyze the forces between the two proteins from a two-dimensional angle. PyMOL was used to map the amino acid residues involved in interactions between the two proteins.

**Determination of cell half-toxic concentration (CC_50_)**

For CC_50_ assay, 3D4/21 cells were seeded in 96-well plates at 1×10⁴ cells/well and incubated until 70% confluency. Cells were treated with serially diluted 9''-methyl salvianolate B (0-120 μM, in ≤0.1% DMSO) for 48 h. Cell viability was measured by CCK-8 (Dojindo, Japan) following manufacturer’s protocol. Absorbance at 450 nm was recorded using a SpectraMax i3x microplate reader (Molecular Devices). CC_50_ values were calculated from three independent experiments (n=6 replicates each) using four-parameter logistic regression in GraphPad Prism 10.0.

**REFERENCES**

1. Cox J, Hein MY, Luber CA, Paron I, Nagaraj N, Mann M. 2014. Accurate proteome-wide label-free quantification by delayed normalization and maximal peptide ratio extraction, termed MaxLFQ. *Mol Cell Proteomics* 13:2513-2526.

2. Livak KJ, Schmittgen TD. 2001. Analysis of relative gene expression data using real-time quantitative PCR and the 2(-Delta Delta C(T)) Method. *Methods* 25:402-408.

3. Hu B, Zhong G, Ding S, Xu K, Peng X, Dong W, Zhou J. 2023. African swine fever virus protein p17 promotes mitophagy by facilitating the interaction of SQSTM1 with TOMM70. *Virulence* 14:2232707.
